# Enhancement of ergothioneine production by discovering and regulating its metabolic pathway in *Cordyceps militaris*

**DOI:** 10.1186/s12934-022-01891-5

**Published:** 2022-08-23

**Authors:** Bai-Xiong Chen, Ling-Na Xue, Tao Wei, Zhi-Wei Ye, Xue-Hai Li, Li-Qiong Guo, Jun-Fang Lin

**Affiliations:** 1 Institute of Food Biotechnology & College of Food Science, South China Agricultural University, Guangzhou, 510640 Guangdong China; 2 Research Center for Micro-Ecological Agent Engineering and Technology of Guangdong Province, Guangzhou, 510640 China

**Keywords:** Antioxidant, Edible fungi, Homologous protease, Biosynthesis pathway, Cordycepin, Fruiting body degeneration

## Abstract

**Background:**

*Cordyceps militaris* is a traditional medicinal fungus contains a variety of functional ingredients and has been developed as an important mushroom food recently. Ergothioneine, one of the antioxidative compounds in *C. militaris*, is benefits on aging-related diseases and therefore became a novel functional food nutritive fortifier. Currently, the main diet source of ergothioneine is mushroom food. However, the mushroom farming faces the problems such as rather low ingredient yield and spontaneous degeneration associated fruiting body that restricts large scale production of ergothioneine.

**Results:**

In this study, we excavated the ergothioneine synthetases in mushroom and modified the genes in *C. militaris* to construct a new ergothioneine synthesis pathway. By further introducing this pathway into *C. militaris* genome, we succeeded to increase the ingredients’ production of engineering strain, the highest amount of ergothioneine and cordycepin were up to 2.5 g/kg dry weight and 2 g/L, respectively. Additionally, the expression of ergothioneine synthetase genes in the shape-mutated degenerative *C. militaris* could recover the ability of degenerative strain to produce high amount of ingredients, suggesting the metabolic regulation of ergothioneine might release the symptom of mushroom degeneration.

**Conclusion:**

This study reveals a new pathway to fulfill the market needs of functional mushroom food and food fortifier ergothioneine. It implied the mycelium of *C. militaris* could be engineered as a novel medicinal mushroom food which could produce higher amount of valuable ingredients.

**Graphical abstract:**

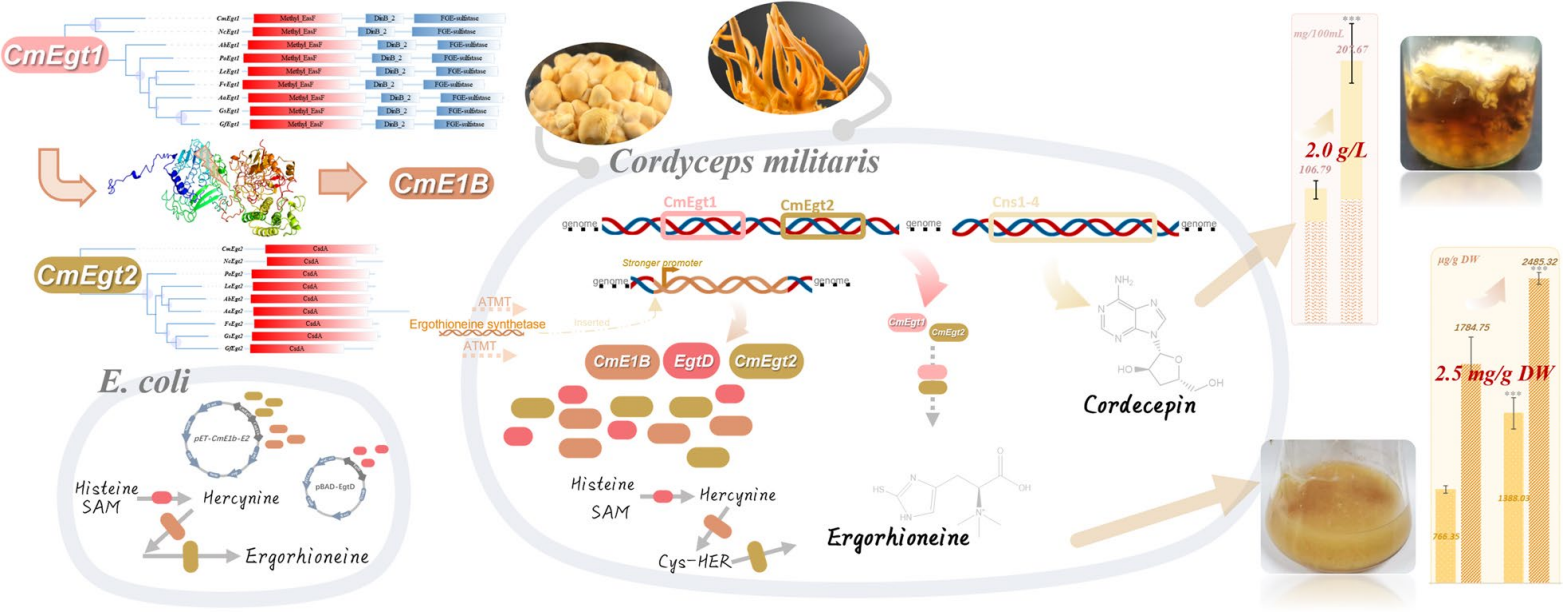

**Supplementary Information:**

The online version contains supplementary material available at 10.1186/s12934-022-01891-5.

## Background

*Cordyceps militaris* is a well-known edible and medicinal fungi for centuries in Asian [1], it has been developed as an important mushroom food recently [2]. It contains many valuable bioactive compounds [3, 4] such as cordycepin and ergothioneine. Ergothioneine, one of the ingredients with great antioxidant and anti-inflammatory activity [5] in *C. militaris *[6], is a natural sulfur-containing thiol molecular and has been regarded as longevity vitamins these years [7, 8]. The specific cationic transport protein (OCTN1) has high affinity for ergothioneine, suggesting the important role of ergothioneine in human physiological activities [9, 10]. But ergothioneine could not be synthesized by plants and animals, including human being. The major diet source of ergothioneine for human is mushroom [11] food, which contains the highest amount (average 150–727 mg/kg dry weight [12]) of ergothioneine than other species in nature. Recently, ergothioneine received self-affirmed Generally Recognized As Safe (GRAS) and approval as a food supplement by the European Food Safety Authority (EFSA) [13], which led it to be a promising new functional food nutritive fortifier.

To obtain higher yield of ergothioneine for the commercial market, engineered strains with different ergothioneine synthetases have been developed in model organism *Escherichia coli *[14–17], *Saccharomyces cerevisiae *[18–20], *Aspergillus oryzae *[21] and et al. [22, 23], but ergothioneine production could only reach 687 mg/L or 231 mg/kg media in their continuous fermentation. The production of ergothioneine in *E. coli* and *S. cerevisiae* can be further increased by strategies of metabolic engineering and fed-batch fermentation, the highest content of ergothioneine were reported to reach 1.31 g/L [15] and 2.39 g/L [20] respectively. However, even with such delicate optimization and high cost in fermentation, the ergothioneine content is not higher enough to fulfill the market needs. As the applications of high-throughput sequencing analysis [24, 25] and genome editing technologies [26] in *Cordyceps*, the genetic breeding strategy can be applicated in *C. militaris* to obtain strain with higher amount of ingredients, therefore *C. militaris* could be developed as a great engineered mushroom host to improve the production of ergothioneine and fulfill the medicinal mushroom food market.

The content of ergothioneine in wild type *C. militaris* was 382 to 799 mg/kg dry weight (DW) fruiting body [27] and 130 ± 11 mg/kg DW mycelia [6], respectively. The rather low content in mycelia indicated the production of ergothioneine in *C. militaris* could be further increased via genetic modification and metabolic engineering. Besides, as one of confirmed factors that caused spontaneous degradation of *C. militaris* is the cellular accumulation of reactive oxygen species (ROS) during the light-induced stage [28, 29], the construction of *C. militaris* strain with overexpression of ergothioneine synthetases is necessary for further studying the degeneration protective function of ergothioneine. However, the biosynthesis pathway of ergothioneine in *C. militaris* has not been discovered yet.

In this study, we identified the ergothioneine synthetases of several mushrooms. The function of *C. militaris* ergothioneine synthetases was verified in *E. coli* with a reconstructed *de novo* pathway. By introducing this pathway into *C. militaris* genome, we succeeded to increase the production of ergothioneine in engineering strains, the highest amount was 2485.32 ± 50.72 mg/kg DW of the 10 days fermentative mycelium. Additionally, we found that the overproduction of ergothioneine in fruiting body degenerated mutant (shape of fat and dwarf) *C. militaris* could recover the high productive ability (approximately 1 g/L) of its main bioactive ingredient cordycepin, while overexpression in wild type could further double the cordycepin content. In conclusion, the discovery and regulation of the ergothioneine metabolic pathway greatly improved the yield of ergothioneine and cordycepin in *C. militaris*, revealing a new way to meet the market needs of functional mushroom food and ergothioneine, and implied that *C. militaris* mycelium could be engineered as a suitable platform to produce higher amount of valuable ingredients.

## Results and discussion

### Conserved domain prediction and sequence alignment of edible mushrooms ergothioneine synthetases

**Fig. 1:**
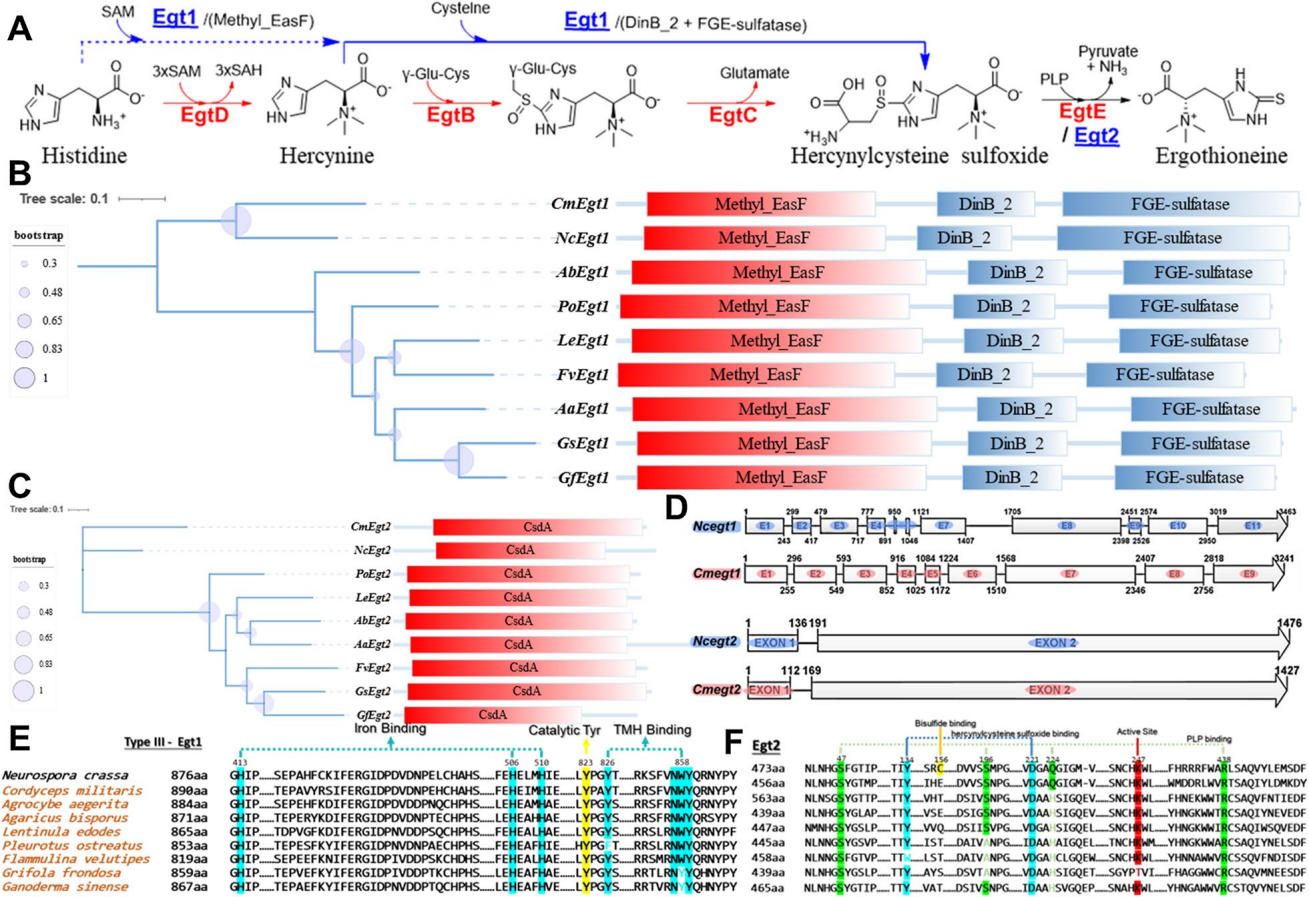
Two different natural biosynthesis pathways of ergothioneine (**A**). A phylogenetic tree with schematic diagram of functional domains contains putative Egt1 (**B**) or Egt2 (**C**) homologous (boxes represented the predicted domains and labeled with corresponding superfamily names). A schematic diagram of genes structure (boxes represented exon and the black line between boxes represented intron) of NcEgt1/2 and CmEgt1/2 (**D**). Sequence alignment of putative Egt1 (**E**) or Egt2 (**F**) homologous in edible mushrooms (predicted key residues for binding were labeled)

Ergothioneine could be synthesized by several pathways [30, 31] in nature (Fig. 1A). These pathways shared similar catalytic route but with different numbers of synthetases (EgtA, B, C, D, E for bacteria [30] and Egt1,2 for fungi [32]). To excavate the biosynthesis pathway of ergothioneine in mushroom, we first analyzed the sequence characterization and functional domain of putative synthetases, which were found by homology sequence analysis. We used the amino acid sequence of NcEgt1/2 as probes to align the sulfoxide synthetases or PLP(pyridoxal 5-phosphatemonohydrate)-depend C-S lyase of ergothioneine with the known genomic sequence of mushrooms (including *C. militaris*, *A. aegerita*, *A. bisporus*, *L. edodes*, *P. ostreatus*, *F. velutipes*, *G. frondosa* and *G. sinense*).

As showed in Table 1, the amino acid sequence of Egt1 from edible mushrooms were showed identification of 33.75–56.43% with the amino acid sequence of NcEgt1, while the amino acid sequence of Egt2 were showed identification of 31.10–48.78% with NcEgt2. They all contained the same functional domains and shared similar sequence length to each other (Fig. 1B, C), which implied the edible mushrooms have a similar ergothioneine biosynthesis pathway with *N. crassa*.

**Table 1:** Putative ergothioneine biosynthesis genes blast from edible mushrooms by comparing with NcEgt1 and NcEgt2

Species	Egt1 - size (aa)	Methyl_EasF^1^ (aa)	DinB_2^2^ (aa)	FGE-sulfatase^3^ (aa)	Query Cover (%)	Per. ident^4^ (%)	Positive (%)	Egt2 – size(aa)	CsdA^5^ (aa)	Query Cover (%)	Per. ident^4^ (%)	Positive (%)
Neurospora crassa	876	36–350	391–514	573–874	100	100	100	473	77–381	100	100	100
*Cordyceps militaris*	890	40–337	417–544	580–887	98	56.43	70	456	72–448	94	48.78	63
*Agrocybe aegerita*	884	21–417	469–598	669–878	93	34.89	47	563	31–420	94	35.65	51
*Agaricus bisporus*	871	20–403	457–586	659–868	95	34.52	49	439	22–430	93	34.80	52
*Lentinula edodes*	865	20–398	452–581	654–862	99	34.37	48	447	29–420	96	33.62	54
*Pleurotus ostreatus*	853	5-381	438–570	640–850	96	34.64	49	445	25–425	94	34.90	52
*Flammulina velutipes*	819	2-362	416–541	611–816	99	35.57	47	458	34–436	96	33.40	51
*Grifola frondosa*	859	27–403	450–579	648–856	97	33.81	50	439	20–339	93	31.10	47
*Ganoderma sinense*	867	27–410	457–586	656–864	98	33.75	48	465	26–455	93	34.96	52

^1^Methyl_EasF: probable methyltransferase domain, EasF family; represents about 300 amino acids with homology to *S*-adenosylmethionine-dependent methyltransferases

^2^DinB_2: DinB superfamily; The DinB family are an uncharacterized family of potential enzymes. The structure of these proteins is composed of a four-helix bundle

^3^FGE-sulfatase: Sulfatase-modifying factor enzyme 1; probable functions as an iron (II)-dependent oxidoreductase

^4^Per. Ident: represent percent identity of amino acid sequence between the input genes to NcEgt1 or NcEgt2

^5^CsdA: CsdA superfamily; probable selenocysteine lyase or cysteine desulfurase

We labeled the key residues for binding and catalysis of the putative Egt1s (Fig. 1E) and Egt2s (Fig. 1F) of edible mushrooms (Additional file 1: Table S1) based on previously identified motifs, which were responsible for enzymic functions [31, 33]. The amino acid sequences of Egt1s in different edible mushrooms were sharing the same iron binding sites and catalytic residues tyrosine. But the hercynine (TMH) binding sites of putative Egt1 genes from *P. ostreatus*, *G. frondosa* and *G. sinense* were distinguish with others, which suggested that these three species might use a different substrate rather than TMH to perform synthesis of hercynylcysteine sulfoxide (Cys-TMH).

Previous study using biochemical and X-ray crystallographic approaches to reveal the NcEgt2 contains a unique bisulfide binding site, but none of Egt2 extracted from edible mushrooms in this study were found to own the similar residues, which implicated that the transsulfuration reaction within ergothioneine biosynthesis pathway in edible mushrooms was different from the reaction in *N. crassa*. Regarding the substrate hercynylcysteine sulfoxide binding site, FvEgt2 showed different residues from others, which could be the reason why the ergothioneine biosynthesis pathway of *F. velutipes* requires two copies of Egt2 [16]. For the binding of cofactor PLP, CmEgt2 contains the same 4 PLP binding sites as NcEgt2, but the other 7 edible mushrooms exist one or two different sites. Furthermore, since GfEgt2 showed a lower identity with NcEgt2 and possessed a different active site with others, it implies that they might share different protein structure and chemical reaction mechanism.

In particular, the putative ergothioneine biosynthesis genes CmEgt1 (accession number CCM_07351) and CmEgt2 (accession number CCM_01645) extracted from *C. militaris* showed the highest identity score (56.43% and 48.78%) with NcEgt1 and NcEgt2. In addition, these genes have similar characteristics of sequence length and exon location (Fig. 1D). All these results implied the ergothioneine synthetases CmEgt1 and CmEgt2 were shared similar catalytic activity and substrate specific with NcEgt1 and NcEgt2, respectively.

### Function determination of ergothioneine synthetases CmEgt1 and CmEgt2

#### The strategy of heterologous expression in *E. coli* with ergothioneine synthetases in *C. militaris*

**Fig. 2:**
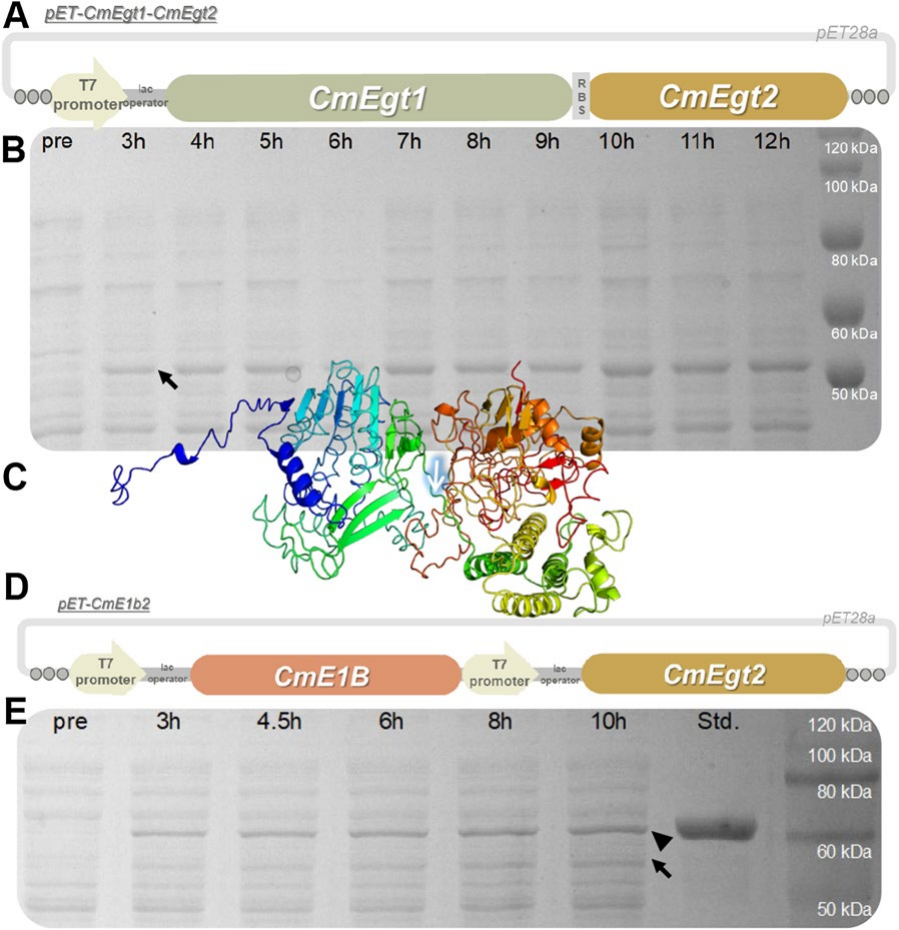
Vector diagram of pET-CmEgt1-CmEgt2 (**A**) and pET-CmE1b2 (**D**). The SDS-PAGE analysis of time course experiment of BL21-pET-CmEgt1_Egt2 (**B**; CmEgt1, 99.4 kDa; CmEgt2_His-tag, 53.3 kDa) and BL21-pET-CmE1b2 (**E**; CmE1b_His tag, 59.5 kDa; CmEgt2_His tag, 53.8 kDa) (Pre, the sample of fermentative cells before induction; 3–12 h, the sample of fermentative cells during the fermentation after induction; triangle and arrow, pointed out the putative CmE1b and CmEgt2 respectively). Predicted protein structure (**C**) of CmEgt1 by I-TASSER (the cut site of truncated protein CmE1b was pointed out)

To verify the ergothioneine synthesis function of CmEgt1 and CmEgt2, we cloned their coding genes from the transcript of *C. militaris *[25], and inserted the genes into *E. coli* expression vector to build pET-CmEgt1-CmEgt2 (Fig. 2A). For monitoring the expression of these proteins, the vector was transformed into *E. coli* BL21(DE3) to perform time course experiment. As result showed (Fig. 2B), the CmEgt2 were expressed after the activation of the promoter by inducer. However, we did not observe the overexpression of CmEgt1. We subsequently performed multiple trials, including changing different expression vectors (including pET-22b from Novagen, Merck Millipore, USA and pCold-I from Takara, Japan) and performing optimization of fermentation, but all of them were failed to optimize the expression of CmEgt1. Even though the SDS-PAGE analysis did not show the soluble expression of CmEgt1, we further performed fermentation of BL21-pET-CmEgt1-CmEgt2 to detect the ergothioneine. Unfortunately, we could not detect any ergothioneine in fermentative medium or sonicated cell pellet via HPLC assay (Additional file 1: Fig. S1).

Previous study indicated the NcEgt1 could be expressed in *E. coli *[32], but CmEgt1 was failed to express in this study. It implicated they may share different protein structure, so we performed protein structure prediction of CmEgt1 with I-Tasser to figure out the problem. As the protein structure showed (Fig. 2C), the CmEgt1 was assembled with three distinguish parts, which contained a putative unstructured signal peptide, a N-terminal *S*-adenosylmethionine (SAM)-dependent methyltransferase domain and EgtB (a nonheme iron-depended sulfoxide synthase that plays role in ergothioneine biosynthesis in bacterial) [34]-like domain. The signal peptides in fungal proteins usually affect the protein expression in *E. coli*. But we have tried to fuse a pelB leader sequence (the inherent peptide of pET-22b, performed function of secretion) to the N-terminal of CmEgt1, the pelB leader could not help to increase the expression of soluble recombined CmEgt1. It implied that the signal peptide of CmEgt1 may not be the only reason of expressing obstacle of CmEgt1.

Previous study [32] also reported that the N-terminal methyltransferase part of NcEgt1 showed a lower catalytic rate than the other part (NcEgt1 could catalyze the substrate histidine or TMH to synthesize Cys-TMH but the reaction rate of using TMH as substrate to perform synthesis was 100-fold fast than using the histidine). As the purpose of this research was constructing a *C. militaris* strain with higher production of ergothioneine, we would rather not to intensive study the function of the N-terminal of CmEgt1 because of its putative low enzymatic efficiency. Moreover, the N-terminal domain could be replaced by a more efficiency isoenzyme such as EgtD (ergothioneine biosynthetic methyltransferase in bacteria).

So, we directly cut off the signal peptide alongside with N-terminal domain at the middle of dividing peptide between the SAM-dependent methyltransferase domain and EgtB-like domain (Fig. 2C, Additional file 1: Table S1) to design the truncated protein CmE1b. Next, the CmE1b, together with CmEgt2, were inserted into the expression vector to build pET-CmE1b2 (Fig. 2D). To increase the production of proteins, the genes of CmE1b and CmEgt2 were driven by two separate T7 promoter respectively. This vector was therefore transformed into *E. coli* BL21(DE3) to perform enzyme co-expression. The time course experiment (Fig. 2E) obviously indicated the overexpression of CmE1b and CmEgt2 and proved our speculation that the signal peptide and/or N-terminal methyltransferase domain could impede the expression of CmEgt1 in *E. coli*.

#### The in vitro reaction verified the ergothioneine biosynthesis function of CmE1b and CmEgt2

**Fig. 3:**
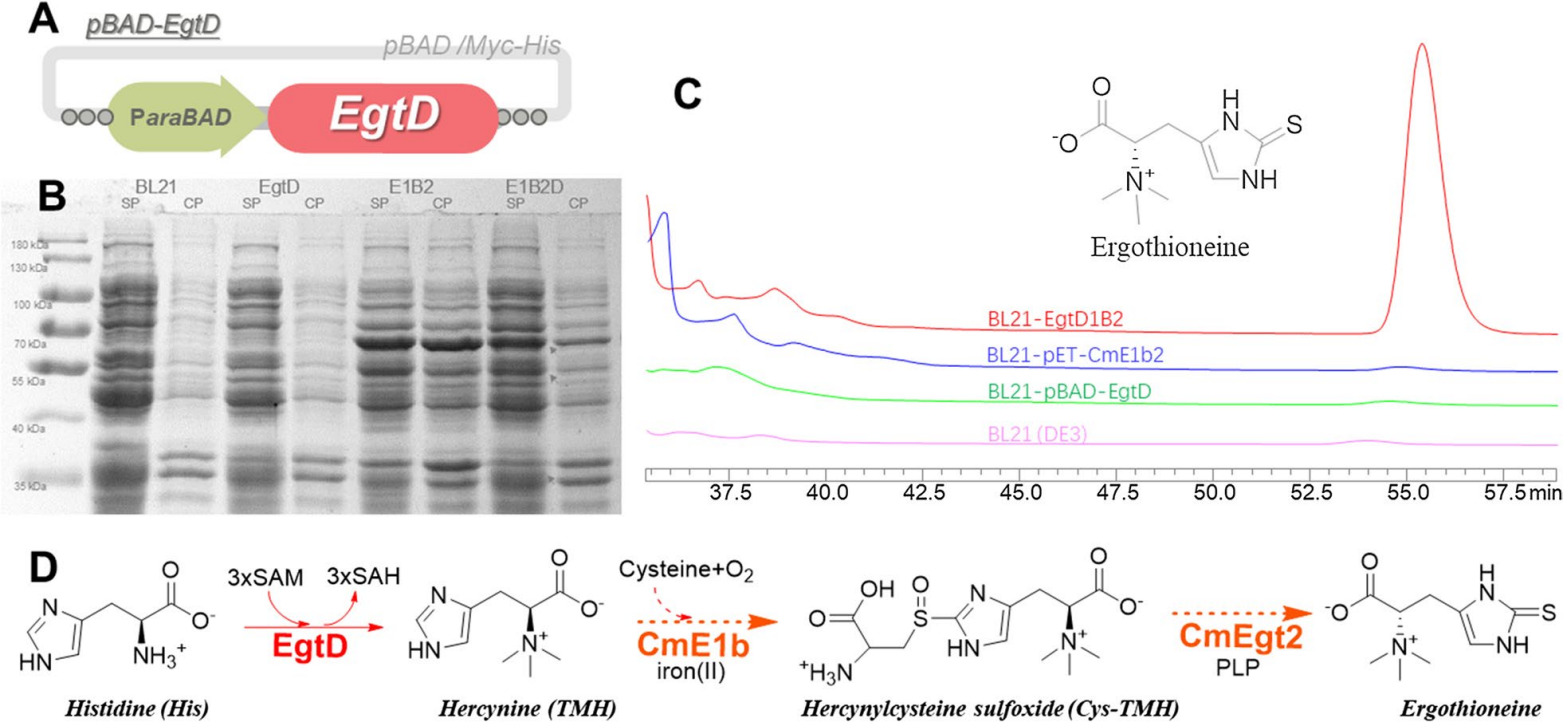
**A** Vector diagram of pET-CmE1b2; **B** SDS-PAGE analysis of protein expression of *E. coli* BL21(DE3), BL21-pBAD-EgtD, BL21-pET-CmE1b2 (E1B2) and BL21-EgtD1b2 (E1B2D). (The supernatant (SP) and cell pellet (CP) of samples after the process of cell sonication and centrifuge; CmE1b, 59.5 kDa; CmEgt2, 53.8 kDa; EgtD, 36.4 kDa; putative proteins were pointed out by arrow); **C** The HPLC analysis (partly chromatogram of the retention time from 36.5 to 58.5 min, all the sample performed at the same condition and showed in same intensity) of in vivo reaction by supernatant from the fermented *E. coli* samples; **D** The putative ergothioneine synthesis pathway of BL21-EgtD1B2

The cut off of the methyltransferase domain of CmE1b suggested the pathway could not perform the conversion of histidine to TMH, so we built a pBAD-EgtD vector (Fig. 3A), which contains the sequence of EgtD with *E. coli* codon optimization, to endow *E. coli* with the ability to synthesize hercynine as reported before [14]. The pBAD-EgtD, coupling with pET-CmE1b2, were transformed into *E. coli* BL21(DE3) to build BL21-EgtD1B2. The fermentation of this strain could simultaneously obtain the proteins of CmE1b, CmEgt2, and EgtD (Fig. 3B). To rapid test the function of CmE1b and CmEgt2, we set up the in vitro reaction and directly processed the fermentative strains with sonication to obtain enzymes for whole cells reaction. As the HPLC chromatogram showed (Fig. 3C), only the samples extracted from BL21-EgtD1b2 could catalyze the substrates (contained histidine, cysteine, and SAM) to synthesize ergothioneine.

In this part, we combined the known function EgtD, which could catalysis histidine to TMH, with putative ergothioneine synthetases CmE1B and CmEgt2 to establish an in vitro ergothioneine synthesis pathway (Fig. 3D). Though the function of N-terminal of CmEgt1 was not verified by overexpression or deletion experiment, its high identical with N-terminal of NcEgt1 implied they both share the similar function of catalyzing histidine to TMH.

### Construct the recombined *C. militaris* strains with high productive of ergothioneine

**Fig. 4:**
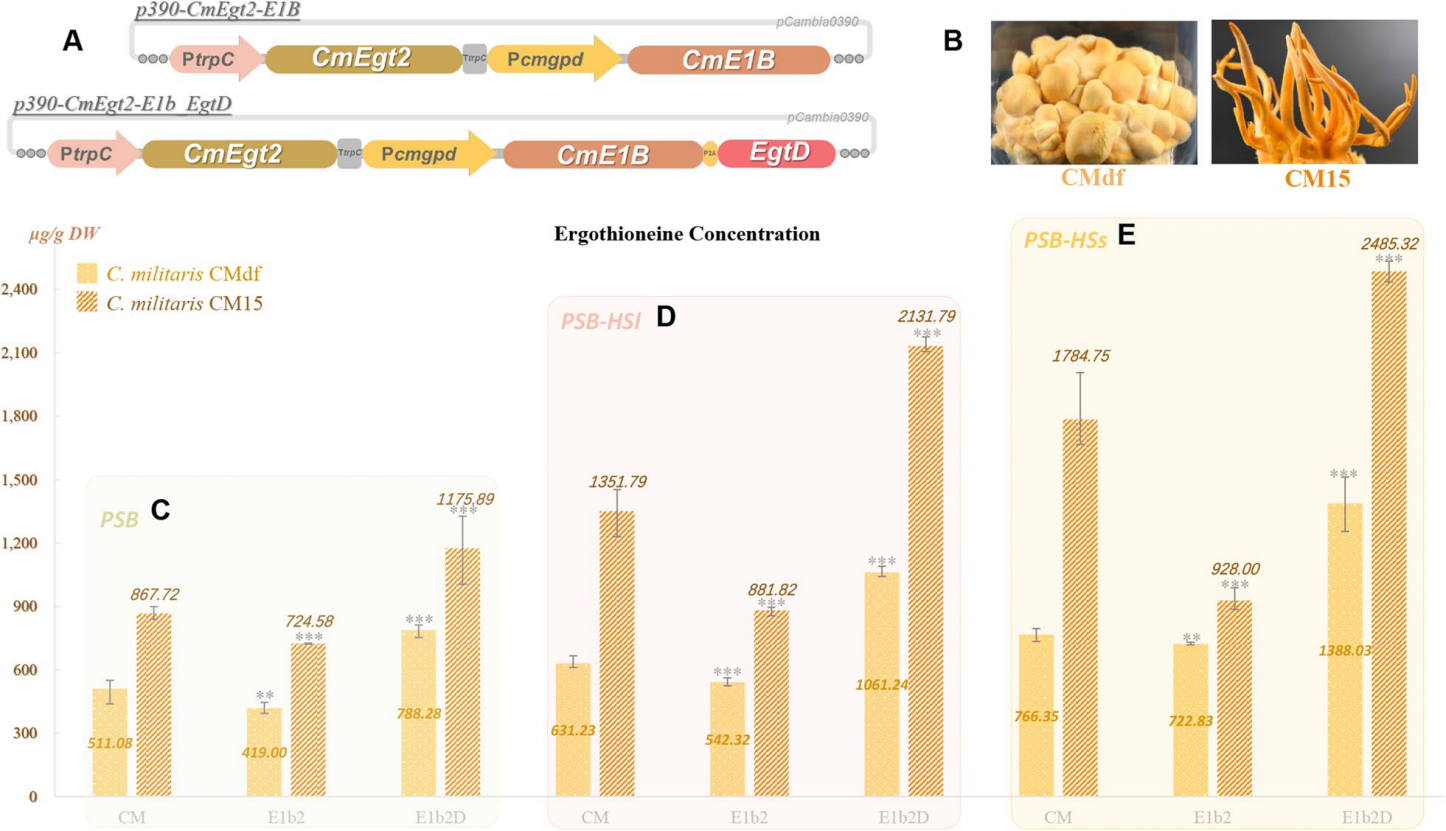
**A** Vector diagram of p390-CmEgt2-E1B and p390-CmEgt2-E1b_EgtD; **B** Mature fruiting body of *C. militaris* CMdf and CM15; The ergothioneine concentration of *C. militaris* fruiting body degenerated mutant CMdf (spot and yellow) and wild type CM15 (slash and orange) and synthetases overexpressed strains (Df-E1b2/15-E1b2: *C. militaris* CMdf/CM15 with overexpression of CmE1b and CmEgt2; Df-E1bD2/15-E1b2D: *C. militaris* CMdf/CM15 with overexpression of CmE1b, CmEgt2 and EgtD.) in PSB (**C**), PSB-HSl (**D**) and PSB-HSs (**E**) media. Statistical analyses were performed using t-tests (**p < 0.001, ***p < 0.0001)

Previous section indicated the assembling of EgtD, CmE1b and CmEgt2 could perform in vitro ergothioneine synthesis, but the bio-function (ergothioneine biosynthesis and degenerative protection) of this new pathway in *C. militaris* is unclear. The construction of *C. militaris* strains, which were carrying the overexpression of ergothioneine synthetases, is therefore necessary for biofunction verification. So, the vectors of p390-CmEgt2-E1B and p390-CmEgt2-E1b_EgtD (Fig. 4A) were constructed and transformed into two kinds of *C. militaris* (contains the normal wild type CM15 and fruiting body degenerated mutant strain CMdf, Fig. 4B) via ATMT (*Agrobacterium tumefaciens*-mediated transformation) method. After several rounds of unselective subculture with the transformants, the colonies were inoculated on the resistance screening plates (Additional file 1: Fig. S2A). The remaining colonies were therefore selected based on the performance of grown rate and pigment accumulation (Additional file 1: Fig. S2B). The qualified recombined strain was further verified by PCR (Fig. S2C) and picked to perform fermentation (Additional file 1: Fig. S2DEF). Finally, we obtained four recombined *C. militaris* strains in total, and they were named 15-E1b2, Df-E1b2, 15-E1bD2 and Df-E1bD2.

These recombined strains were inoculated in PSB medium (Fig. 4C, Additional file 1: Fig. S2G), which were optimized to increase the ergothioneine production of edible mushrooms with sucrose as carbon source, to perform flask fermentation of ergothioneine. After 10 days cultivating, the mycelium of CMdf and CM15 produced 511.08 ± 39.69 and 867.72 ± 31.88 mg/kg DW of ergothioneine, respectively. The production were corresponding or higher than the strains (contains *S. cerevisiae *[18] and *A. oryzae *[21]) with heterologous expression of NcEgt1 and Egt2s. It indicated the potential of building *C. militaris* as an ergothioneine high productive host. In addition, the overexpression of CmE1B, CmEgt2, and EgtD in recombined *C. militaris* strains (Df-E1b2D or 15-E1b2D) could perform a 1.36 to 1.54 times (than CMdf or CM15) increase of ergothioneine production in the fermentation with PSB media (Fig. 4C). Consider the precursor shortage always be the main problem of metabolite production, we supplemented the substrates (histidine and SAM) in PSB media before the fermentation. As the result showed (Fig. 4D and E), the production of ergothioneine were all increased. The highest amount was up to 2485.32 ± 50.72 mg/kg DW, which were produced by 15-E1b2D with PSB-HSs medium (contained 0.3 mM histidine and SAM in PSB). It suggested that the bacteria source EgtD, CmE1B and CmEgt2 could be over-expressed and constructed a high yield ergothioneine synthesis pathway in *C. militaris*.

Besides, compared the ergothioneine yield of strains in PSB-HSs and PSB-HSl (contained 3 mM histidine and SAM in PSB), they showed that the excessive substrates may decrease the yield of ergothioneine, which suggested that the production of ergothioneine could be further optimized by starting from the adjustment of the concentration of additives.

Meanwhile, the ergothioneine production of Df-E1b2 and 15-E1b2 showed obviously decreased (compared to CMdf or CM15). It suggested the biosynthesis of ergothioneine in *C. militaris* was not limited by CmE1b and CmEg2. The overexpression of them resulted in a decreasing of ergothioneine implied they may join in other unknown reactions that compete with ergothioneine precursor. In other words, it means that the synthesis of the precursor substrate TMH was the rate-limiting step to synthesize ergothioneine in *C. militaris*.

In this section, we constructed two expression boxes which contained the coding genes for ergothioneine synthesis (contained CmE1B, CmEgt2, and EgtD), and inserted them into the genome of *C. militaris* CMdf/15 for the purpose of breeding the ergothioneine high productive recombined strains. We obtained up to 2533.28 mg/kg DW of ergothioneine in PSB-HSs flask fermentation of *C. militaris*. So far, the fruiting body of edible mushrooms is the major diet source of ergothioneine for human being, because it usually contains the higher concentration of metabolites than the mycelium. But the fruiting body always faces a serious degeneration problem, which restricts the development of industrial bioactive ingredient extraction and medicinal mushroom food. The engineering overexpression strategy in this study revealed that the molecule breeding of *C. militaris* could engineer mycelium as a suitable platform to promote the yield of high-value ingredients.

### Endogenous synthesized ergothioneine highly promote the cordycepin production of degenerated *C. militaris*

The continuous cultivation of *C. militaris* always along with high frequency degeneration, which represented as fruiting bodies distortion and secondary metabolites reduction. Previous research indicated that the ROS accumulation was one of the main factors to induce degeneration in *C. militaris *[28, 29]. Although the degenerate phenotype of *C. militaris* is irreversible, we speculated that the relief of ROS accumulation would recover the property of producing the main metabolites. Because the ergothioneine was reported to prevent the ROS over-accumulated in fungi cells [35], we investigated the differences of morphology and metabolites yield among wild type CM15, degenerative mutant CMdf and their recombined strains with ergothioneine synthetases (15-E1b2, Df-E1b2, 15-E1bD2 and Df-E1bD2) in *C. militaris*.

**Fig. 5:**
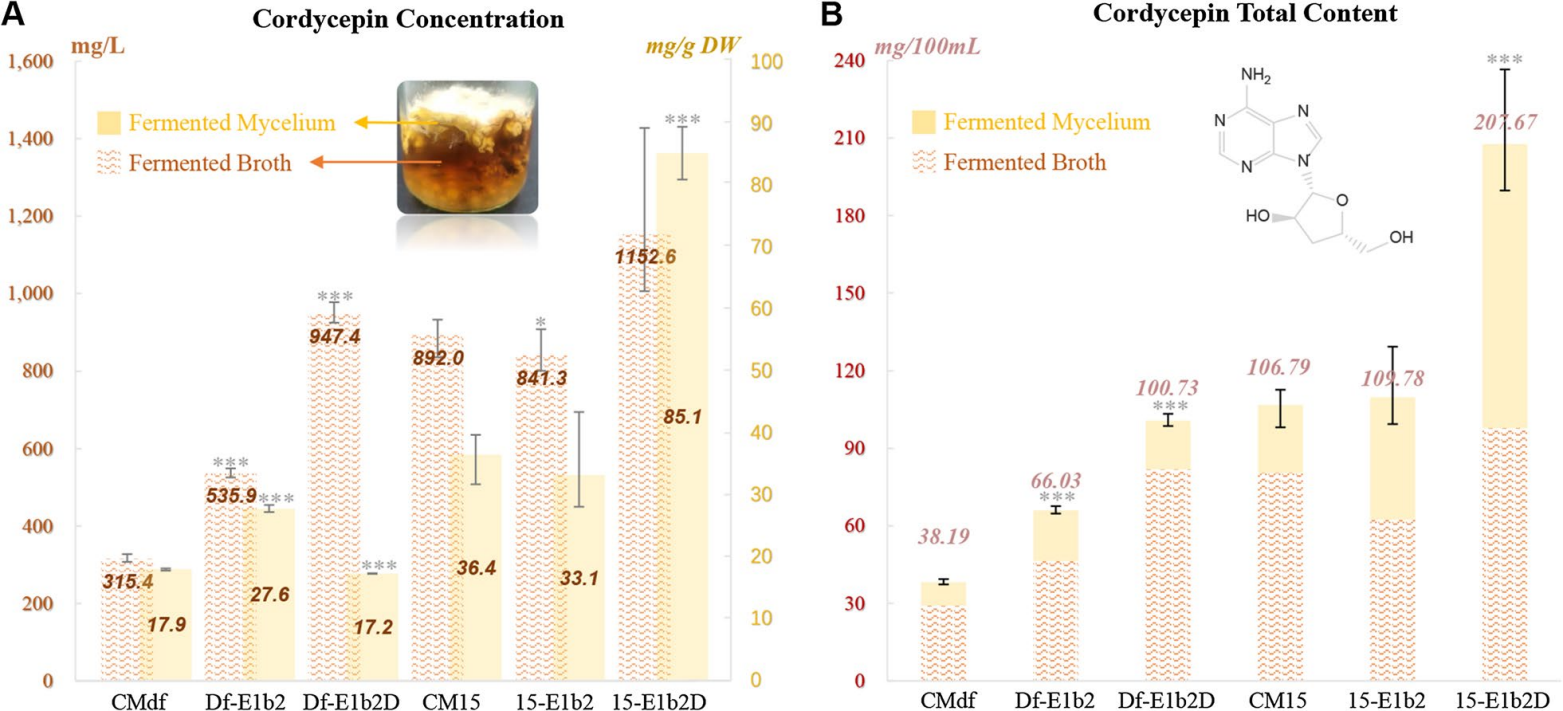
The cordycepin concentration (**A**) and the total content (**B**) of *C. militaris* wild type and its derived in AM medium after 30 days static submerged fermentation. Statistical analyses were performed using t-tests (*p < 0.01, **p < 0.001, ***p < 0.0001)

As Fig. 5A showed, the wild type *C. militaris* CM15 could produce 0.89 g/L of cordycepin in AM medium, while CMdf only produced 0.32 g/L of cordycepin. The lower yield of cordycepin of CMdf showed that the degeneration of CM15 will change the shape of the fruiting body, and perform a significant reduction in metabolite production, which consisted of the pattern of the *C. militaris* degeneration [28].

In addition, the cordycepin content (Fig. 5B) of 15-E1b2 showed that overexpression of CmE1B and CmEgt2 in healthy wild type (compared to CM15) could barely affect the cordycepin yield. But the concentration of cordycepin in Df-E1b2 had a double amount increasing (compared to CMdf). It indicated these two genes were not directly relevant to the production of cordycepin, but they were roles of joining the metabolic regulation of degenerated strains.

The results (Fig. 5) showed that the overexpression of CmE1B, CmEgt2 and EgtD obviously improved the cordycepin production, while the increasing patterns were different between wild type CM15 and shape mutant type CMdf. As Fig. 5A showed, the synthetases overexpression promoted a triple increase of cordycepin content in fermented broth (compared with samples of CMdf and Df-E1b2D), while the concentration in the mycelium barely changed. But the cordycepin concentration of 15-E1b2D was increased 1.29-time (in broth) and 2.34-time (in mycelium) than CM15. As result, the total cordycepin amount of 15-E1b2D (Fig. 5B), which reached 0.2 g per grass jar (contained 100 ml of AM medium), was double than its original strain CM15. However, the fermentation of Df-E1b2D performed just equal amount to that of in CM15. It showed that the cordycepin yield of the shape degenerated strain could be raised, but the in vivo cordycepin production will face increasing bottleneck. Regarding to the antioxidant ability of ergothioneine, it also implied that the change of ROS environment might be one of the reasons for the decrease in cordycepin yield in the shape degenerative strain. In summary, the increasing content of cordycepin indicated the ergothioneine synthetases played roles in the excretion or production of cordycepin.

Previous report [29] showed that the generation of *C. militaris* fruiting body could be recovered by introducing the antioxidase Gpx in genome of degenerative strain. It suggested that the antioxidant ability of *C. militaris* might also be important to fruiting body germination. We therefore performed fruiting body farming with wild types and mutants to further study the relationship between ergothioneine and fruiting body germination. The experiment was different of previous study [29], the degenerated strain of this study have the ability of generating fruiting body. We aimed to test the ability of recover the fruiting body shape from dwarf to long. Unfortunately, the morphology of the fruiting body between recombined strains and its original wild type did not show difference. Though the ergothioneine content of CMdf was increased, the ability of forming healthy slender fruiting body was failed to recover. It indicated the fruiting body mutant degeneration in *C. militaris* might be irreversible, but the amount of metabolites in mutant strains could be increased back to normal level.

## Conclusion

We excavated the ergothioneine synthetases Egt1 and Egt2 of 8 micro fungi by bioinformatic homologous alignment and verified the ergothioneine synthesized function of CmE1B, CmEgt2, and EgtD in *E. coli*. By constructing this new pathway in *C. militaris* wild type, we increased the yield of ergothioneine and cordycepin by up to 2.5 g/kg DW and 2 g/L, respectively. Though the overexpression of these synthetases in degenerated *C. militaris* strain failed to recover the morphology of fruiting body back to normal, the improving ingredients concentration implied the potential of developing *C. militaris* as chassis cells for producing high amount of ergothioneine, and breeding a functional mushroom food to fulfill market needs.

## Methods

### DNA manipulation and strains construction

Vectors and strains used in this study were listed in Table 2. The coding sequence of putative ergothioneine synthetases (Additional file 1: Table S1) in *C. militaris* were amplified from total RNA of *C. militaris* CM15 [25] by Phanta Max Super-Fidelity DNA polymerase (Vazyme Biotech, Nanjing, China) with default conditions. All the vectors used in this study were constructed by overlap-PCR and ClonExpress II One Step Cloning Kit (Vazyme Biotech, Nanjing, China). Primers and detail constructed methods were list in Additional file 1: Table S2. The backbone vector used for the construction of synthetases overexpression in *C. militaris* was built before [26]. It contains two expression boxes which were under controlled by strong promoters PtrpC and Pcmgpd respectively. Briefly, we assembled two vectors, the first vector called p390-CmEgt2-E1B, which contained the CmEgt2, controlled by a medium strength PtrpC promoter, and the CmE1b, promoted by a strong strength Pcmgpd promoter. The other one called p390-CmEgt2-E1b_EgtD, which contained the coding gene of EgtD (Additional file 1: Table S3). In particularly, the EgtD coding sequence was located at the end of previous vector’s CmE1b open reading frame by a 2 A peptide linker [36]. The *C. militaris* strains were constructed with these two vectors by ATMT method as previous described [26]. The wild type of strain with inserting the expression boxes of CmE1b and CmEgt2 was named 15-E1b2, while the degenerated fruiting body mutant strain with the same insertion was named Df-E1b2. Similarly, the wild type CM15 with inserting the CmE1b_EgtD (coding genes of Egt2 and CmE1b were linked together) and CmEgt2 was named 15-E1bD2, while the degenerated mutant CMdf with the same insertion was named 10-E1bD2.

**Table 2:** Vectors and strains used in this study

Names	Description	Original plasmid or strain	Source
*Plasmid*			
*p390-blpR-sgRNA-cmcas9-gfp*	Agrobacterium-mediated transformed vector with TrpC promoter, Cmgpd promoter, glufosinate-ammonium resistant selection marker Blp^R^, Km^R^	–	[26]
*pET-CmEgt1-CmEgt2*	Carrying CmEgt1 and CmEgt2 at *NcoI* and *XhoI* sites, these genes were drove by one T7 promoter and separated by ribosome bind site (RBS), Km^R^	*pET28a*	Lab collection
*pET-CmE1b-E2*	Carrying CmE1b and CmEgt2 at *NheI* and *XhoI* sites, these genes were severally droved by different T7 promoter, Km^R^	*pET28a*	Lab collection
*pBAD-EgtD*	Carrying EgtD at *SacI* and *HindIII* sites, Cm^R^	*pBAD-Myc-His*	Purchase from Thermo fisher Scientific, MA, USA
*p390-blpR-CmEgt2*	Inserting the CmEgt2 into *XbaI* site, to build the expression cassette PtrpC-CmEgt2-TtrpC, Blp^R^, Km^R^	*p390-blpR-sgRNA-cmcas9-gfp*	[26]
*CmE1b_EgtD*	Linking CmE1b and synthesized EgtD [30] with a self-cleaving 2 A peptide [36] to build cassette CmE1b-P2A-EgtD	Synthesized	Lab collection
*p390-CmEgt2-E1B*	Inserting the CmE1b into *PstI* and *BcuI* sites, to build the expression cassette Pcmgpd-CmE1b-Tnos, Blp^R^, Km^R^	*p390-blpR-CmEgt2* and *CmE1b-EgtD*	This study
*p390-CmEgt2-E1b_EgtD*	Inserting the CmE1b_EgtD into *BcuI* site, to build the expression cassette Pcmgpd-CmE1b_EgtD-Tnos, Blp^R^, Km^R^	*p390-blpR-CmEgt2* and *CmE1b-EgtD*	This study
*Strain*			
BL21-pET-CmEgt1_Egt2	Carrying the vector *pET-CmEgt1-CmEgt2*, Km^R^	*E. coli* BL21 (DE3)	Lab collection
BL21-pET-CmE1b2	Carrying the vector *pET-CmE1b2*, Km^R^	*E. coli* BL21 (DE3)	Lab collection
BL21-pBAD-EgtD	Carrying the vector *pBAD-EgtD*, Cm^R^	*E. coli* BL21 (DE3)	Lab collection
BL21-EgtD1b2	Carrying the vectors *pET-CmE1b2* and *pBAD-EgtD*, Cm^R^, Km^R^	*E. coli* BL21 (DE3)	Lab collection
*Cordyceps militaris* CM15	Wild type strain, with slender and long fruiting body	*-*	Purchase from Shandong, China
*Cordyceps militaris* CMdf	Degenerated strain of CM15 which was breeding by continued cultured and showed dwarf and oval shape fruiting body	*Cordyceps militaris* CM15	Lab collection
*15-E1b2*	With overexpression boxes of CmE1b and CmEgt2 from p390-CmEgt2-E1B in genome of CM15, Blp^R^	*Cordyceps militaris* CM15	Lab collection
*15-E1b2D*	With overexpression boxes of CmE1b and CmEgt2 from p390-CmEgt2-E1b_EgtD in genome of CM15, Blp^R^	*Cordyceps militaris* CM15	Lab collection
*Df-E1b2*	With overexpression boxes of CmE1b and CmEgt2 from p390-CmEgt2-E1B in genome of CMdf, Blp^R^	*Cordyceps militaris* CMdf	Lab collection
*Df-E1b2D*	With overexpression boxes of CmE1b and CmEgt2 from p390-CmEgt2-E1b_EgtD in genome of CMdf, Blp^R^	*Cordyceps militaris* CMdf	Lab collection

### Fermentation conditions

The protein overexpression of *E. coli* was performed in Lysogeny Broth (LB) and supplemented with corresponding antibiotic and cofactors. The precultures for overexpression cultivation were prepared at 37 ℃ with 200 rpm agitation for 16 h. The fermentation of *E. coli* was performed as follow. Seed culture were inoculated in LB media and cultivated at 37℃ until OD600 was 0.8, 0.2 mM IPTG or/and 0.5% l-arabinose was/were supplemented and continue cultivated at 20 ℃ for 12 h. The overexpression of proteins was verified by SDS-PAGE electrophoresis. In particularly, the samples of time course were collected by each hour while induced and cultivated for three hours.

*C. militaris* was inoculated in 100 ml PDB media and cultivated at 25℃ with shaking for 5 to 8 days as seed culture of ingredients fermentation. Ergothioneine fermentation of *C. militaris* was performed in potato sucrose broth (PSB, contains 200 g/L potato, 20 g/L sucrose, 4 g/L NH_4_Cl, 3 g/L KH_2_PO_4_ and 1.5 g/L MgSO_4_·7H_2_O; PSB-HSl, supplementary with 3 mM 0.3mM histidine and SAM in PSB; PSB-HSs, supplementary with 0.3mM histidine and SAM in PSB), which could induce *C. militaris* to produce higher yield of ergothioneine compared to several common macro-fungi cultivation media. 10% of seed culture were inoculated in PSB and cultivated at 25 ℃ for 4 days with shaking in dark, then cultivated with light for 6 days. Cordycepin fermentation were also begun with 10% of seed culture and performed in AM media at 25 ℃ for 30 days as previously described.

### Detection and quantification of cordycepin and ergothioneine

After fermenting in AM media, the mycelia were dried off at 50 ℃ and ground to powder. Two hundred milligram of dry powder was weighted and immersed in 20% of methanol to perform ultrasonication. Then, the supernatant of media and the crushing fluid were collected to perform HPLC detection as previously method [25]. The detection of ergothioneine from *C. militaris* fermentative samples were performed as follow. The mycelia were collected and immersed in 70% of ethanol. After treating by ultrasonication, the supernatant was used for HPLC detection while the precipitation was dried off to obtain the wight of original mycelia. HPLC assay was performed by Ultimate HILIC Amphion II Column (4.6 × 250 mm, 5-Micron, Welch, Shanghai, China). The analysis conditions were as follow: mobile phase, 10% ultra-pure water and 80% acetonitrile (v/v); flow rate, 0.9 ml/min; detection wavelength, 259 nm; column temperature, 30 ℃. A standard ergothioneine curve was generated using 5–60 mg/L ergothioneine standard (Sigma-Aldrich, United States). The yield of cordycepin and ergothioneine were calculated using the detected peak area according to the standard curve. The cordycepin or ergothioneine concentrations of mycelia and/or fermentative media presented in the study were calculated by normalizing in the equal biomass.

### Statistical and bioinformatic analysis

The experimental data were represented as mean ± standard error of mean of three replicates. For graphical representation and analysis Microsoft Excel were used. The yield of cordycepin or ergothioneine between two strains were compared based on biological triplicates and significant difference was identified with p-value < 0.01. The protein structure prediction was performed by uploading the amino acid sequence into I-TASSER On-line Server [37] (https://zhanggroup.org/I-TASSER/) with default setting. The sequence alignments were performed by using the online software BLAST^41^ in NCBI (https://blast.ncbi.nlm.nih.gov/Blast.cgi) and MEGA (version 7.0, https://www.megasoftware.net).

### Enzymatic reaction assay

Since the enzyme kinetics of ergothioneine synthetase EgtD [38, 39], EgtB [34], NcEgt1 [32] and NcEgt2 [33] had been studied. We performed enzymatic reaction with ultrasonicated whole cell to verify the ergothioneine biosynthesized function of CmEgt1 and CmEgt2 rather than using purified enzyme because of the overexpression of CmEgt1 in *E. coli* faced obstacles. After fermenting in LB for 12 h, the collected cells were diluted in Tris Buffer (100 mM Tris, 150 mM NaCl, pH8.0) to perform sonicating. After using high speed centrifuge to separate the cell pellet, all the supernatants were performed enzymatic reaction immediately. The reaction was set up at 25 ℃ for overnight with stirring and contained the following substrates: 1 mM histidine, 3 mM *S*-adenosylmethionine (SAM), 1 mM Cysteine, 1 mM dithiothreitol and 100 mM ascorbic acid. Last, the reacted samples were lyophilizate and filtrated before submitting to HPLC detection.

## Supplementary Information

**Additional file 1:** **Table S1**. Amino acid sequence and domains prediction of fungi ergothioneine synthetases. **Table S2**. Primers and detail constructed methods of vector construction in this study. **Table S3**. DNA sequence of synthesized EgtD. **Fig. S1**. The HPLC chromatogram of ergothioneine detection of the fermentative medium and cell pellet of BL21-pET-CmEgt1_Egt2. **Fig. S2.** The verification of qualified recombined *C. militaris* strains.

## Data Availability

All data generated or analysed during this study are included in this published article [and its supplementary information files]. The materials used and/or constructed during the current study are available from the corresponding author on reasonable request.

## Abbreviations

DW: Dry weight
ROS: Reactive oxygen species
PLP: Pyridoxal 5-phosphatemonohydrate
His: Histidine
TMH: Hercynine
Cys-TMH: Hercynylcysteine sulfoxide
SAM: *S*-adenosylmethionine
