# Supplementary material for: Enhancement of ergothioneine production by discovering and regulating its metabolic pathway in Cordyceps militaris
Source: Microb Cell Fact. 2022 Aug 23;21:169. doi: 10.1186/s12934-022-01891-5 (PMC9396837; doi:10.1186/s12934-022-01891-5)
Supplement: Supplementary file 1 — Additional file 1: Table S1. Amino acid sequence and domains prediction of fungi ergothioneine synthetases. Table S2. Primers and detail constructed methods of vector construction in this study. Table S3. DNA sequence of synthesized EgtD. Fig. S1. The HPLC chromatogram of ergothioneine detection of the fermentative medium and cell pellet of BL21-pET-CmEgt1_Egt2. Fig. S2. The verification of qualified recombined C. militaris strains. [file 12934_2022_1891_MOESM1_ESM.docx]

**Enhancement of ergothioneine production by discovering and regulating its metabolic pathway in *Cordyceps militaris***

Bai-Xiong Chen ^a,b^, Ling-Na Xue ^a,b^, Tao Wei ^a,b^, Zhi-Wei Ye ^a,b^, Xue-Hai Li ^a,b^, Li-Qiong Guo ^a,b,^*, Jun-Fang Lin ^a,b,^*

^a^ Institute of Food Biotechnology & College of Food Science, South China Agricultural University. Guangzhou, Guangdong, 510640, China;

^b^ Research Center for Micro-Ecological Agent Engineering and Technology of Guangdong Province, Guangzhou, 510640, China.

^*^ Co-corresponding author: Professor Li-Qiong Guo and professor Jun-Fang Lin, Tel: +86-20-85285382; E-mails: guolq@scau.edu.cn (L-Q Guo) and linjf@scau.edu.cn (J-F Lin).

**Additional file 1**

Table S1. Amino acid sequence and domains prediction of fungi ergothioneine synthetases.

**Table S2**. Primers and detail constructed methods of vector construction in this study.

**Table S3**. DNA sequence of synthesized EgtD.

**Fig. S1**. The HPLC chromatogram of ergothioneine detection of the fermentative medium and cell pellet of BL21-pET-CmEgt1_Egt2.

**Fig. S2.** The verification of qualified recombined C. militaris strains.

Table S1. Amino acid sequence and domains prediction of fungi ergothioneine synthetases

| ***Notes***  **>’Self-defined Name’: ‘NCBI Protein Definition’ – ‘Accession Number’ [‘Species’] ‘Amino Acid Length’**  **Red front:** Methyltransf_33 superfamily: **Histidine-specific methyltransferase, SAM-dependent;** The mycobacterial members of this family are expressed from part of the ergothioneine biosynthetic gene cluster. EGTD is the histidine methyltransferase that transfers three methyl groups to the alpha-amino moiety of histidine, in the first stage of the production of this histidine betaine derivative that carries a thiol group attached to the C2 atom of an imidazole ring.  **Green front:** DinB superfamily: The DinB family are an uncharacterized family of potential enzymes. The structure of these proteins is composed of a four-helix bundle.  **Blue front:** FGE-sulfatase super family: Sulfatase-modifying factor enzyme 1; This domain is found in eukaryotic proteins required for post-translational sulfatase modification (SUMF1). These proteins are associated with the rare disorder multiple sulfatase deficiency (MSD). The protein product of the SUMF1 gene is FGE, formylglycine (FGly), -generating enzyme, which is a sulfatase. Sulfatases are enzymes essential for degradation and remodeling of sulfate esters, and formylglycine (FGly), the key catalytic in the active site, is unique to sulfatases. FGE is localized to the endoplasmic reticulum (ER) and interacts with and modifies the unfolded form of newly synthesized sulfatases. FGE is a single-domain monomer with a surprising paucity of secondary structure that adopts a unique fold which is stabilized by two Ca^2+^ ions. The effect of all mutations found in MSD patients is explained by the FGE structure, providing a molecular basis for MSD. A redox-active disulfide bond is present in the active site of FGE. An oxidized cysteine residue, possibly cysteine sulfenic acid, has been detected that may allow formulation of a structure-based mechanism for FGly formation from cysteine residues in all sulfatases. In Mycobacteria and Treponema denticola this enzyme functions as an iron(II)-dependent oxidoreductase.  **Orange front:** Selenocysteine lyase/Cysteine desulfurase |
| --- |
|  |
| >NcEgt1: DUF323 domain-containing protein egt-1 - XP_956324 [*Neurospora crassa* OR74A] 876aa |
| *MPSAESMTPSSALGQLKATGQHVLSKLQQQTSNADIIDIRRVAVEINLKTEITSMFRPKDGPRQLPTLLLYNERGLQLFERITYLEEYYLTNDEIKILTKHATEMASFIPSGAMIIELGSGNLRKVNLLLEALDNAGKAIDYYALDLSREELERTLAQVPSYKHVKCHGLLGTYDDGRDWLKAPENINKQKCILHLGSSIGNFNRSDAATFLKGFTDVLGPNDKMLIGVDACNDPARVYHAYNDKVGITHEFILNGLRNANEIIGETAFIEGDWRVIGEYVYDEEGGRHQAFYAPTRDTMVMGELIRSHDRIQIEQSLKYSKEESERLWSTAGLEQVSEWTYGNEYGLHLLAKSRMSFSLIPSVYARSALPTLDDWEALWATWDVVTRQMLPQEELLEKPIKLRNACIFYLGHIPTFLDIQLTKTTKQAPSEPAHFCKIFERGIDPDVDNPELCHAHSEIPDEWPPVEEILTYQETVRSRLRGLYAHGIANIPRNVGRAIWVGFEHELMHIETLLYMMLQSDKTLIPTHIPRPDFDKLARKAESERVPNQWFKIPAQEITIGLDDPEDGSDINKHYGWDNEKPPRRVQVAAFQAQGRPITNEEYAQYLLEKNIDKLPASWARLDNENISNGTTNSVSGHHSNRTSKQQLPSSFLEKTAVRTVYGLVPLKHALDWPVFASYDELAGCAAYMGGRIPTFEETRSIYAYADALKKKKEAERQLGRTVPAVNAHLTNNGVEITPPSSPSSETPAESSSPSDSNTTLITTEDLFSDLDGANVGFHNWHPMPITSKGNTLVGQGELGGVWEWTSSVLRKWEGFEPMELYPGYTADFFDEKHNIVLGGSWATHPRIAGRKSFVNWYQRNYPYAWVGARVVRDL* |
| **>NcEgt2: aminotransferase - XP_001728131.1 [*Neurospora crassa* OR74A] 473aa** |
| *MVATTVELPLQQKADAAQTVTGPLPFGNSLLKEFVLDPAYRNLNHGSFGTIPSAIQQKLRSYQTAAEARPCPFLRYQTPVLLDESRAAVANLLKVPVETVVFVANATMGVNTVLRNIVWSADGKDEILYFDTIYGACGKTIDYVIEDKRGIVSSR****C****IPLIYPAEDDDVVAAFRDAIKKSREEGKRPRLAVIDVVSSMPGVRFPFEDIVKICKEEEIISCVDGAQGIGMVDLKITETDPDFLISNCHKWLFTPRGCAVFYVPVRNQHLIRSTLPTSHGFVPQVGNRFNPLVPAGNKSAFVSNFEFVGTVDNSPFFCVKDAIKWREEVLGGEERIMEYMTKLAREGGQKVAEILGTRVLENSTGTLIRCAMVNIALPFVVGEDPKAPVKLTEKEEKDVEGLYEIPHEEANMAFKWMYNVLQDEFNTFVPMTFHRRRFWARLSAQVYLEMSDFEWAGKTLKELCERVAKGEYKESA* |
|  |
| >CmEgt1: CCM_07351 DUF323 domain protein - XP_006672555 [*Cordyceps militaris* CM01] 890aa |
| *MPAVKEAFYAQPVAVSHAVTTGDAATPRKMPTTQQQHLDIIDIRGEQVDFNLKEEIAGCLNPVSGHRTLPTLLLYDEKGLQLFEDITYLDEYYLTNYEIELLTKHAAEIANLIPAGSMVVELGSGILLSHGRNLRKISLLLDAFEKIAKSIDYYALDLSEDELRRTLLQIPDYKHVRSHGLWGTYDDGKDWLKQFANSDRDITIIHLGSSIGNFSRNEAGSFLQGFAQVMKPTDSVIIGIDSCCDPNKPRVQWQVDIEALARLKLTLNAADSKGVTHSFILNGLRHANTIHGSEIFHLDDWKVIGEFIYDEDGGRHQAFLSPTKEVTILGTTIKQAERLKIEQSLKYSQVGGEKLWRTAGLKEVKRWRRGDDYGLHLLQKTDLPFSSLPSQYASVLPTLKDWSALWQAWDIVTKGMLPGQELQEKPIKLRNACIFYLGHIPTFLDIQLSKVTGEPATEPAVYRSIFERGIDPDVDNPEHCHAHSEIPEEWPPVDEIAAYQGRVRARLETLYSNSNNGDPDKMPRSVARGIWVAFEHEIMHIETLLYMMLQSDRTLPPPHTPMPDFAALGAAARRARVPNAWVNVPEQTVTLGMNDPEDGTDPTVNFGWDNEKPERKQKVHAFQAKARAITNEDYADYLYSTKNGTLPASWSTVAEAHRNSPILNGAGSEYPMQPYLKDKVVRTVYGLVPLKYALDWPVAASYNELAGCAAWMGGRIPTMEEAKSIYHFAEKQKEAAMHSKLSNRVPAVNGHLVNNGVEETPPDSTAKPAVNGSGGSYETSLYIDLDGANVGFQTWHPQPVTARGDTLAGQAGMGGVWEWTSSPLTRHAGFEPMSLYPAYTSDFFDGKHNVVLGGSWATHPRIAGRRSFVNWYQRNYPYAWAGARLVRDVA*  **CmE1b:** *LPSQYASVLPTLKDWSALWQAWDIVTKGMLPGQELQEKPIKLRNACIFYLGHIPTFLDIQLSKVTGEPATEPAVYRSIFERGIDPDVDNPEHCHAHSEIPEEWPPVDEIAAYQGRVRARLETLYSNSNNGDPDKMPRSVARGIWVAFEHEIMHIETLLYMMLQSDRTLPPPHTPMPDFAALGAAARRARVPNAWVNVPEQTVTLGMNDPEDGTDPTVNFGWDNEKPERKQKVHAFQAKARAITNEDYADYLYSTKNGTLPASWSTVAEAHRNSPILNGAGSEYPMQPYLKDKVVRTVYGLVPLKYALDWPVAASYNELAGCAAWMGGRIPTMEEAKSIYHFAEKQKEAAMHSKLSNRVPAVNGHLVNNGVEETPPDSTAKPAVNGSGGSYETSLYIDLDGANVGFQTWHPQPVTARGDTLAGQAGMGGVWEWTSSPLTRHAGFEPMSLYPAYTSDFFDGKHNVVLGGSWATHPRIAGRRSFVNWYQRNYPYAWAGARLVRDVA* |
| >CmEgt2: CCM_01645 cysteine desulfurylase, putative - XP_006666863.1 [*Cordyceps militaris CM01*] 456aa |
| *MTVTNQTDLPTRGKYLEFGGQLKTLFPQAQDWVNLNHGSYGTMPLVIREKFRAYQDLAEATPDKFIRYDQGKLIDESREAVAKLVNAPTDTVVFVTNATEAVNTVFRNMKWNEDGKDVILFFSTIYPACAKIADFMVDYFGSHRVGIHEIPLHYPLEDEDIIQLFRDAVAALEKQGKRARICTFDVVSSNPGLVFPWEALCKACKELDVLSMVDGAQGIGMVELDLAAADPDFFTSNCHKWLHVPRGCAILYCPLRNQDMIATPLSTSHGYVPRTAVRRDVLPPNTKPPFVNRFELVATKDRSQDIVTKDAIAWRRDVCGGEARIMAYLWDLNKRGSRHVAARLRTEVLENTKGTLTNCAMANIALPIWLPGGKGKGAGEHEEDMVVPEHEAVQVRLWMMQTMKDEYHTLMPLFWMDDRLWVRTSAQIYLDMKDYEYAAKVLEELAARVANGEYKI* |
|  |
| >AaEgt1: unnamed protein product - CAA7262981 [*Agrocybe aegerita*] 884aa |
| *mydvirnrsscrvginaplsatmpieiidihshsndgiivsdipqqilaglslppgqrqmptmllyderglrlyddittevpeyylfgaeeeilknkadeivrtmhrgvevrsdevivelgagalrktshilaglsrlmgevqpstpityyaldleqrelertlngiarsevgeqligkihtkgmcgtyddglkfiesgalfsqnvsddlnssdelnsqdaspfssdfsvsdassetdsptistpegsniplhimflgsslgnfsrsgaasflkglplrpgsgdtlllgldhdnekdrieeayndkqgftrrfifnglkaagralgnpdmfdegnweyvnhynvaerrheaffkakcahtvsdpvthktisfmkdelmkieesikfsetdtytmfteanlrvvqrwmdsksqyslwllerpplifptlsaslaadqvprkystspfgvpsreewkhlwaawdfitlrmippsmlfqrpidlrhiclfylghiptfldihlskllnephsepehfkdifergidpdvddpnqchphsevpaededwptlttilkfqrvvrerlmklyddidsgkicltrkvgrvlfmtleheamhaetllymllqrsgtgtlpppdfilppwaslaaawdetplpssatitlgpetialgqddieaedgvldgdarthsfgwdnespkrsvrvgqfriewrpvtngqfyqfykdggkeqvefpsswveeagevkirtvydpvpmyiahdwpviasyeslstyaivkggrlptepelrlfydkfesgyegganvgfrnwhpipattggernggkginggvwewtsttfdavdgfvpsklypgysmdffdqkhqvvlggsyatipriferrsfrnwyqrnypypwvggrvvydvesk* |
| > AaEgt2: unnamed protein product - CAA7265062.1 [*Agrocybe aegerita*] 564aa |
| MGDVDYKALYTQRPPPFGEELKAYFALDPEYINLNSGSYGTTPKPVLQAAYELTKKIEANPDLFHRFTYQPMLIDVRKRIARLIGAKTDEVVLVTNASLGLNIVLRNFDWEEGDAIFAFTTTYNSISRTAQSIGDVPPHPTVHTIPLNFPTTHEEIASSFKEFLRAHRVAPNKKRVAIIDSIVSNPGVLLPWQEMVKIAKEERVWSVVDAAHSIGQEVGINLTESAPDFWISNNHKWLSAKRSSAVLYVPGRNQHIVTTSIPTSYAYVSPKDRTEPNFVPQYEWNGTIDWSSHLTVADALDFRTWLGGEAKINAYCHNLALNGGKILAEILDTRVMDPDGDLTLNMINVELPLPPVTTIDEYNRIDVFLKERLLTKYKAYSAHFFHNEKWWTRCSAQVFNTIEDFEKIGRIWVKACEEVKNGLHLKYMSVISIHSAGEIQVQIDENCRRLCYRRANSVSSSPRQTPRPATHAPILALYAQDPTRATRAFTALHRTDPAEAGALLGALFLAGDDSRRVGRFLLQSGGLFAQGDRGMVDVLERAMRWCLGRSQEPRPAYLGFSSV |
|  |
| >AbEgt1: hypothetical protein Agabi119p4_7241 - KAF7767998 [Agaricus bisporus var. burnettii] 871aa |
| mpaeivqlhshstsspftnieqlvheglshpigeknlptillyderglklydkittqaaeyypfqaeeqilkrsaddivrmmhgsisgpvadevvlelgagalrktslllaalarlidndssnfpityyaldlerselertlgdieqsplgnmlqdkvetkgicgtyddglkflseggfrgahivgslpdftdkitnttltrnppsadssdsrdsrstsafstlpsspgdpavspfhilflgsslgnfpradavkflrglplrsgygdtlligldhdndtalvekayndtqghttefimnglraagrvlgneelfneknweyvnkydakerrheaflkakvdhsinspggnvyvfkadellkieesfkfsygdatslfteaglrpiqrwvdnnsryslwlverppfvfplmsssfscnrsgelvpkkaystspfgipareewdnswalwdfitlrmiprsmlhekpihlrhiclfyighipafldiqlsrllnephteperykdifergidpdvdnptechphsivptkteewptlaeilrfqesvrsrvmsiyddiesgkrllsrqlgrllfmtleheahhaetllymllqragtgtipppgfaepdwqslaaawdaeprpeseivtlgpemvsighddleaddektskvsepdhheygwdlehperklevqqfkiswrpvtngefyefylgqegdkielpaswvveddvvrvrtiygpismdlarhwplmtsydnlsayamvkggrlptepelrlfydkfnsgyegganvafrnwhplpattggakndgkghnggvwewtsteldrvdgyvpsklypgysndfmdgkhhvvlgasyataprvserrsfrnwyqrsypyawiggrivydk |
| **>AbEgt2: hypothetical protein AGABI2DRAFT_178673 - XP_006461570.1 [*Agaricus bisporus var. bisporus* H97] 439aa** |
| *MAMRDISPDELFKRDPPPFGRPMLDYFPLDPEYINLNHGSYGLAPYIVHKAAQDLSFKLEANPDRFLRLECLDYLNDVRQRLANLVKVDRDEIVLVPNTSVGVNTILRNFEWEKDDVIICFNTTYNSVYQTACNLGDIPPYPTVSEIKLGFPTTPHQIITQFRDHIKTLALQRKDTSKKSTKSPKCVAIIDSIGSNPGVYLPWKEMVKICKEEGIWSVVDAAHSIGQEQDIDLRSVEPDFWVSNCHKWLHCKKSVAMLYIPERNRDIIKTSLPTSHAYRPVKDRSLRDFLAQFEWNGTIDFIPYLTVGTALDFRAWIGGEAKIFEYCHNLAIEGGKRMAEILGTRVMDPNGEFTLNMVNVELPLPGRILWSSQVKTMLDEKMLNQRNAYSAHFYHNGKWWTRCSAQIYNEVEDFEKLAKIWIEVCDEVTREVDGGNGKP* |
|  |
| >LeEgt1: hypothetical protein HHX47_DHR4000834 - KAF8827866 [Lentinula edodes] 865aa |
| *MPAEIVDVQTSENLRAIQLQILDGLQRPAGQKNLPTMLLYDERGLRLYDDITTMAPEYYLFGAEEDILKKHATDIVMAMHNTTGCIPGETVIELGAGSLRKTSHILRSLSHIVDSPSRVPPITYYALDLEKRELERTLNAIAISDIGSDLKGKVETKGMWGTYDDGLKYLKSTGLYAPTAIDRPSRLEASMRFDARDLSPSSTTSGSDSSGAHVFDVSPPSTPEEVQAPLHIMFLGSSLGNFNRKDGAAFLHSLPLRPGSGDTLLLGLDHANEKDLIEEAYNDPRNHTKKFIMNGLRGAGRALGNETLFLEDKWDYVNRYDDIERRHEAFYKSKISQVLRIPDGDISVEFQENELVKVEESWKFSETDAYTLFSESNLRPIQRWIDSTSRYSLWLLERPAFIFPLLKSPNACNSSSELVPKKTYSSSPFGVPSPQEWENLWAAWDMITLRMIPPSMLFQKPIDLRHICLFYLGHIPTFLDIHLSRLLSEPHTDPVGFKDIFERGIDPNVDDPSQCHPHSEVPQNDKDWPSLNSILQFRSRVRNRLLKLYNDFDNGNLTLTRKMGRVLFMTFEHEALHAETLLYMLLQRAGSGTIPPHGFAIPEWNSLASSWKSIPPLVEDTVTLGPAVVELGHDDAEGEDELKDCKFNVEDREFGWDNEHPKRHVDVQEFRISWRPITNGQYYDTFKKNKDKFHVPASWVEEDGEIRVRTLYGPVPLSVAEDWPIFASYDDLSTYATVKGGRLPTEPELRLFYDKFQCGFEGGANLGFRNWHPVPATTGGKQNGGKGHNGGVWEWTSTEFDKYDGFTPSKLYPGYSMDFFDGAHQVVIGGSYATIPRIAERRSLRNWYQRNYPFAWVGARVVYDA* |
| > LeEgt2: PLP-dependent transferase - KAH7876913.1 [Lentinula edodes] 447aa |
| *MANVVSQYESKPPPFGHEMRKCFSFDPEYINMNHGSYGSLPSPVAEAIKPYYALAEANPDLFHRFTSIDLIRDVRKRIAKFIGAAHTEEVVFVPNASHGLNTILRSFIWEEGDIISSCNTTYNSISRTAEYISDIPPHPEVVQFTLLFPTSHSEIIQNWRAYVRSLNEMRDQSAQKLGRRPKIVAIIDSIISVPGALLPWKEMVKICKEEDVWSVVDGAHSIGQELDLNLAEANPDFWVTNCHKWMYAKRGCALLYVPKRNQHIIKASFPTSHAYKKGGGSNFVEQFEWNGTIDFVPYISASCALDFRAWLGGEHVINDYCHRIALEGGKRLAQILGTEIIDQDQDFQFTLSMVNVAVPFPPTMSSAFEIDMAFRRKLLIKRKIYPAFFYHNGKWWIRCSAQIWSQVEDFEVLGQAILEASKEILEEFGDGTEKKTMGIEDKVEKVN* |
|  |
| >PoEgt1: hypothetical protein PLEOSDRAFT_1058188 - KDQ26018 [*Pleurotus ostreatus* PC15] 853aa |
| *Maiqivdvqtlnqsrhldlqsdlsqqlvdgltrphgekeiptvllyderglrlydaittevseyylfgaeeeilknkadeivrtmhsglgdadpdsevvlelgagslrktshilaglsrivpsecdtapityyaldleerelqrvldsisnssvgemlrgkvetkglwgtyedglkfvedegspisshsnsptsqssrrelgppsptprsgsssplhilflgsslgnfdrkdsvkflqsmplrpgsgdtlligldhdndktlieeayndrkgytktfimnglraagralgnedmfeedkweyvnryneaerrheayykskcpqkledpkaehgyefledelvkievsykysetdayslftdsglrpiqrwtdsatqyslwllerppfmfpllkspiafnglgeivknfplsntpfgvpspqewsnlwaawdfvtlkmippsmlhvkpihlrhiclfylghiptfldihlsrllkephtepesfkyifergidpnvdnpaechphsevpqnegdwpsleailsfqarvrsrllnlyddvltgkkvltrkvcrilsmtfeheafhietllymllqaagngttpppgftpppwsslsanwnalpalenttvtlgpeivalghddderededpafitdvkdhefawdnenpkrqvevkqfkiewrpvtngqfyefykkhkddmklqlpaswletddkmmvrtlygpvpmkvaqnwpvvisydglsayaivkggrlptepelrlfldkfesgyegganvgfrnwhpvpattggkkyggkghnggvwewtsttltkhdgfepskhypgftadffdgchnvilggsyafiprmaerrslrnwyqrnypyawtggrivydi* |
| >PoEgt2: hypothetical protein PLEOSDRAFT_1113034 - KDQ26326.1 [Pleurotus ostreatus PC15] 445aa |
| *MNFELDEVCRTSPPPFGHAMLKYFGFDPKYVNLNNGSYGSVPLPVHAECNKLTLDVERNPDFHHRFTHYDMQAQVRQRVAPFIGAQADECVIVPNATHGINTVLRNIEWENGDIIIDFNTTYGAVSRTAQYLSDRPPHPSISTLTINFPSTHADIISKFRAHVKSVVDSKPPGKRAVALIDAIVANPGVRLPWQELVKICKEADVLSVIDAAHAIGQELNINLGEAQPDFWVSNCHKWLFSKRGCAVLYVPRRNQHLIKTSIPTSHSYVSPGNPPTFIEQFNWNGTMDFVPFYSVLPGELIIRGVRVRLHAYLLALRFREWLGGEDKINAYCHDLAIAGGKRLAEVLGTQVLDPDGSLTLNMVNVEIPLSGEIKWTPEIKNRFSEKLLKEHNVFAATFYHNGKWWARCSAQIYNELSDFKFIGKAFLQICPEIQQEFGVDSVATV* |
|  |
| >FvEgt1_revised: unpublish sequence from Lab stored Transcriptome data [*Flammulina velutipes* YX74] 819aa |
| *MLLYDEQGLRLYDAITTEAPEYYLFGAEEEILKTRADDIVRVMHAGAGLSAGEVVLELGSGALRKTSHVLRSLAGLVANSNLSGSSPITYYALDLEYRELERTLGDIASSDLGPILKDKVSTRGMWGTYEDGLKFLQSGGLHSPSPSSQMAAMGRHLSREFDSADRDNSPDSRSSASSTDAGSSPPSTPGETQQPLHLLFLGSSLGNFTRAEGAEFLRSLPLRPGMGDTLLLGLDHDNDQRVIEEAYNDPRGHTEAFIVNGLKAAGRVLGDDKLFDGGKWEYVNKYDVETRCHEAFFKCKEDYQVQREDALFTFVKDELVKIEQSLKFSDNDAYALFSDSGLRPIQRWTDSASRYSLWLLERPPFLFPLLSSPVACNAQGQITPKKIYSTTPFGIPSPQDWENLWAFWDFITRSMIPPSMLFQKPIDLRHICLFYLGHIPTFLDIHLSRLLKEPHSEPEEFKNIFERGIDPIVDDPSKCHDHSEVPTKESDWPSLASILQFQSRVRKRVLDVYGGVKTIDRKTARVLFMTFEHEALHAETLLYMLLQRAGSGTLPPAGFSPPDWTSLKVQWDALSKPAESSVTLGPAIISLGHDDFEAEDATASDAREHEFGWDNEHPKREVPVGQFRIEWRPITNGEFYSFYRSKEGEGMELPTSWVEGDGGIQVRTLYGPVSMDIARDWPVIATYDALSRYATVKGGRLPTEPELRLFYDKFESGYEGGANVGVRNWHPVPSTTGGGRGGNGGVWEWTSTVFDDYEGFKASPLYPGYSKDFFDGKHNVVLGGSYVTIPRIAGRRSMRNWYQRNYPYAWVGGRVVYDM* |
| >FvEgt2: PLP-cysteine desulfurase - QBB19874.1 [*Flammulina velutipes*] 458aa |
| *MVHHSWSMDYRAGFYDSTHPPPEFGHAMLKYFSLEPGYVNLNNGSFGTVPRPVTRFCNELTNKVEANPDRYHRFSFKPLLADSRERVAQLVGAHTDECVFVSNATAAINTIMRNLEWNAEDIIIQTTTTWRSTSRVMHYIADTRPYPMLSTFKLTFPTTHKAILEAFHAHIKGLKNNMLTVSENGRQVRKGKIVAVIDAIVANPASYMPWKDMVAICREEGVVSLVDGAHCLGQEVGINLSEIRPDFWISNCHKWLYSRRGSAVLYVPFRNQHLIRSSVPTSTHYVSPRDGLGGPKAPNFVMQHEWPGAIDFSSYLCIPTSLEFRAWLGGEEVINTYCRNLSIKGSKRLAAMLGTNVMDETPDSQLTLNMSNVKLPLPANVSNDKKTEVNDFLELSLLREYNVFAVTFYHNNAWWVRCSSQVFNDISDFEKLGTALIALCREVKMSVLNSHGEDATHA* |
|  |
| >GfEgt1: Gfegt1 (Yu, Y.-H., Microb. Cell Factories,2020,19,164) [*Grifola frondosa*] 859aa |
| *MSTLQDFFHIVDLRANQPTLASSVIHEQVVSGLSQPAGQKWLPTMLLYDERGLRLYDAITTEAPEYYLFPAEEEILKNRSSDIVRVMHARNGNAESVEEVVVELGAGALRKTSHILRALSQHSMSSVQYYALDLEKRELERTLKTLHDSEIGAEIKDKVSTKGLCGTYDDGLKFIAEGGLEGRNDLERITTEVSEQYKLERVGGDDSPRSASSSRTPTTETDVTPPSTPGFNQPLHILFLGSSLGNFTRGEDAAFLRSLPLRPGSGDTLLLGLDHDNEAHQIELAYNDPKGITKNFIMNGLKCAGRALGDEHLFDEDKWEYVAMYNEELRRHEAYYKSTCEQTVVDTKTKKCLPFEADELVRIEVSYKFSERDAYTLFTDANLRPIQRWMDSAGQYSLWLLERPKFTFPLLRSPSAIDEKGVVSSPFGMPAMDEWHTMWAAWDFITRQMIPPSMLFQKPIDLRHICLFYCGHIPAFLSIHISKLLEEPDTEPVEFKYIFERGIDPIVDDPTKCHPHSEVPQHDEDWPSLGSILEYQSRVRERVMKLYRDIQSGKVTLTRKIARVLFMTLEHEAFHAETLLYMLLQRAGTGTLPPTGFSPPVWSVLAESWERLPAPHTPTVTLGPETLTVGHDDSEADDNTTDVAGHEFGWDNEHPKRTVHVPEFKIEWRPVTNGEFYEFYIGEGKEQVQLPASWVEIDGEMLVRTFYGPVPMKVAKDWPVITSYDNLSTYASVKGGRIPTEPELRLFLDKFECGYEGGANIGFRNWHPIPATMGGVKDGRGHNGGVWEWTSTVFEKHDGFVPSKLYPGYSMDFFDTHHQIVIGGSYATIPRLAERRTLRNYYQHNYPYAWVGARIAYDV* |
| >GfEgr2: hypothetical protein A0H81_07972 - OBZ72541.1 [Grifola frondosa] 439aa |
| *MTAIDLAAPFGHALKPYWAFDPKYINLNHGSYGSLPLPVLFSCTQNTILAERNPDKFHRVTYMPMLQESRKRVAELVGAEHDEIVLVPNATHGLNTVLRNFEWKQGDVIIGASTTYGAISRTIQYLADRSEQPRPEAYSIQYTFPMSHAEILDAFRARVREIKQLHASTEFSDAPLESLGYEEGSKKNKFVAVIDSVTANPGVLMPWKEMVRVCRKKAFWSVVDAAHSIGQETNINSAKRGLISGYPTVISGFTRNGAVPLYMCPNVIKQALQFRDGYDTNFVLQHEWTGTMDFIPYLSIPAALDFRNWLGGEAAINEYCHELAMAGGERLASVMGTKVMDKTGELTLNMTNVLLPLPVENTKGEVYSGEVLSAINSQLREKLLYEWNTYAAHYFHAGGWWCRCSAQVWNEESDFEYLGKAFNAICKEIKDTLLAEKRN* |
|  |
| >GsEgt1: hypothetical protein GSI_15117 - PIL22429 [Ganoderma sinense ZZ0214-1] 867aa |
| *masngpanavriidiraraepfpgssirdqilsglaqpvghktlptlllyderglriydeittdaseyylfpaeeeilknkadeivrimhagvpdadvmdevvvelgagalrktslilsafarlvpnhssvppinyhaldlekrelertlmelnaseigiqlqgrvttaglcgtyddglkfieeggledrasldpidtslaskysverigrdaspssatsshsrteeteatppstpgsqqplhilflgsslgnfsrgedaaflkslplrpgsgdtlllgmdhgndtkqieaayndakgitkkfimngltcagralgdehlfdedkweyvgtyneelrrheayyratcdqavvdpetktqfpfvkdelirvelshkfserdaytvfaganlrpvhrwtdsssqyslwllerpqfsfpllkmpstldsttavrspfslptveewrdmwaawdfvtrqmipasmlfekpidlrhiclfycghipaflsihlsrllqepdtepaefkyifergidpnvddptqchphsevpqkdedwpslssilqfqsrvrervmnlyrdidsgkvqltrkiarvlqmtleheafhvetllymllqragtgtippagfipppwevlaeswdmqslpttetvtlgpaeislghdddevhdhstdvldhslgwdnenpqrtvqiekfriewrpvtngqfyefyaghgkgkvqfpkswvelngevfirtlygpvpmkiaqhwpiktsfdnlsvyanvkggriptepelrlfldkfecgyegganigfrnwhpvpattggthdggkghnggvwewtstvfekhegfvqsklypgystdffdthhqaviggsyatiprlaerrtvrnyyqhnypyawvgariayda* |
| >GsEgt2: hypothetical protein GSI_02403 - PIL35673.1 [Ganoderma sinense ZZ0214-1] |
| *MPIPKLTPAANETYDTNEKPPPFGHPLKAYFAIADDYVNLNHGSYGTIPLPLIFKVNQWTYEIESNPDLFHRFTYRPLLAKSREALANLIGAEADEVVFAPNATHALNTILRNFEWRDGDVLVGASTTYPAVANTLTYLSDRSESPRPEVATVTLDFPLTHAQIVDIFRAKLREVKQEHPNSQFTDVPPFTPGFSEDGKGKGNKIVAVVDSIVSNPGVLLPWQEMVRVAHEEGVWTVIDAAHSVGQEPNINLTEAKPDFWLSNAHKWLYAKRGCAVLYTPRRNQHIIKSSIPTSQQYISPKSPQWETKGTNYVGQHEWTGTQDWTSFITVPEAIAFRQWLGGEKPIYDYCHQLAIDGAKRLAEILGTRVLDESGELTTTMSNVQLPLPTKDEKPINVDIYWEVDAYLKDKLLFQRKAYAAHFYHNGAWWVRCSTQVYNELSDFERLGKALNEACKEVKDTILSKY* |

Table S2. Primers and detail constructed methods of vector construction in this study.

| **Vectors or DNA sequences** | **Methods** | **Templates** | **Sequence-F** **(5'-3')** | **Sequence-R(5'-3')** |
| --- | --- | --- | --- | --- |
| *CmEgt1* | PCR | *C. militaris* CM15 transcript | ATGCCTGCCGTCAAGGAGGC | AGCGACATCGCGCACAAGGC |
| *CmEgt2* | PCR | *C. militaris* CM15 transcript | atgaccgttaccaatcagaca | aatcttatactccccattggca |
| **pET-CmEgt1-CmEgt2** | Overlap-PCR, *Nco*I, *Xho*I, ClonExpress II One Step Cloning Kit (Vazyme, China) | pET28a,  *CmEgt1*,  *CmEgt2* | TAAGAAGGAGATATACCATGGATGCCTGCCGTCAAGGAGGC | tctccttcttaaagttaaacaaatatctagagcctaAGCGACATCGCGCACAAGGCG |
|  |  |  | tgtttaactttaagaaggagaatgAGGTCGACGATTatgaccgttaccaatcagaca | cagtggtggtggtggtggtgctcgagaatcttatactccccattggc |
| **pET-CmE1b-E2** | Overlap-PCR, *Nhe*I, *Xho*I, ClonExpress II One Step Cloning Kit (Vazyme, China) | pET-CmEgt1-CmEgt2 | TGCCGCGCGGCAGCCATATGGCTAGCCTCCCTTCGCAGTATGCAAGTGTTCTGCCCACACTCA | TCCGCTCACAATTCCCCTATAGTGAGTCGTATTAGAATTCgctttgttagcagccggatc |
|  |  |  | CACTATAGGGGAATTGTGAGCGGATAACAATTCCTCTAGAtatttgtttaactttaagaa | CAGTGGTGGTGGTGGTGGTGCTCGAGTGCGGCCGCAAGCTTaatcttatactccccattggca |
| **pBAD-EgtD** | *Sac*I, *Hin*dIII, T4 DNA Ligase | pBAD/Myc-His,  *EgtD*(synthesized) | - | - |
| p390-blpR-CmEgt2 | *Xba*I, T4 DNA Ligase | p390-blpR-sgRNA-cmcas9-gfp (Front. Microbiol. 2018, 9, 1157), *CmEgt2* | AAGCATCGATtctagaatgaccgttaccaatcagacagatcttccc | TATTAAATCAtctagaCTCAGTGGTGGTGGTGGTGGTGCTCG |
| *CmE1b_EgtD* | PCR, Overlap-PCR | p390-blpR-CmEgt2,  *P2A*(synthesized),  pET-CmE1b-E2 | ATCAAGAACAaCTAGTATGGGCAGCAGCCATCATCATCATCATCACAG | AGCGACATCGCGCACAAGGCG |
|  |  |  | GCCTTGTGCGCGATGTCGCTGGCTCCGGCGCCACCAACTT | TAGTTCGCCAGAGACAGAGTAGAagagccTTTTTCGAACTGCGGGTGG |
|  |  |  | ACTCTGTCTCTGGCGAACTATCTGGCAGCTGAT | tagcgttaacACTAGTGCGCACAGCCAGGGACAGACCA |
| **p390-CmEgt2-E1B** | PstI, BcuI, T4 DNA Ligase | p390-blpR-CmEgt2,  *CmE1b_EgtD* | ATCAAGAACAgCTAGTATGGGCAGCA | tggtggtggctagcgttaacACTAGTcatTGTACATGGGCCGGGGTTCT |
| **p390-CmEgt2-E1b_EgtD** | BcuI, T4 DNA ligase | p390-blpR-CmEgt2,  *CmE1b_EgtD* | ATCAAGAACAaCTAGTATGGGCAGCAGCCATCATCATCATCATCACAG | tggtggtggctagcgttaacACTAGTcatTGTACATGGGCCGGGGTTCT |

Table S3. DNA sequence of synthesized EgtD.

| >synthesized EgtD (codon optimization for *E. coli*) |
| --- |
| *ATGAGCTCGACTCTGTCTCTGGCGAACTATCTGGCAGCTGATTCCGCTGCTGAAGCACTGCGTCGCGATGTTCGTGCGGGTCTGACCGCTGCTCCTAAATCCCTGCCTCCGAAATGGTTCTACGACGCTGTTGGCTCTGACCTGTTCGATCAGATTACGCGTCTGCCGGAGTATTATCCGACTCGTACTGAAGCCCAAATCCTGCGTACTCGTTCTGCTGAAATCATTGCTGCCGCGGGTGCCGACACTCTGGTTGAACTGGGTTCCGGCACTTCTGAAAAGACCCGTATGCTGCTGGACGCAATGCGTGACGCTGAACTGCTGCGTCGTTTCATTCCGTTCGACGTAGATGCTGGTGTTCTGCGTTCTGCTGGTGCAGCTATCGGTGCGGAATATCCGGGTATCGAAATCGACGCTGTATGCGGTGACTTCGAGGAGCACCTGGGCAAAATCCCGCATGTTGGCCGCCGTCTGGTGGTTTTCCTGGGTTCCACGATTGGTAATCTGACTCCTGCACCGCGTGCAGAATTCCTGAGCACTCTGGCAGATACCCTGCAACCGGGTGACTCCCTGCTGCTGGGTACCGATCTGGTAAAAGACACCGGTCGTCTGGTTCGCGCTTACGATGATGCAGCTGGCGTAACCGCGGCATTTAACCGTAACGTTCTGGCAGTGGTTAACCGTGAGCTGTCTGCGGACTTCGACCTGGACGCGTTCGAGCATGTTGCCAAATGGAACTCTGATGAAGAACGTATTGAAATGTGGCTGCGTGCTCGTACTGCGCAGCATGTACGTGTGGCCGCTCTGGACCTGGAAGTTGATTTCGCGGCGGGTGAAGAAATGCTGACGGAAGTCTCTTGTAAATTCCGTCCGGAAAATGTTGTTGCAGAACTGGCGGAAGCAGGCCTGCGTCAGACTCATTGGTGGACCGACCCGGCCGGTGACTTTGGTCTGTCCCTGGCTGTGCGCTGTACATCTAGACATCATCATCATCATCATTGA (EgtD 6xHis)* |


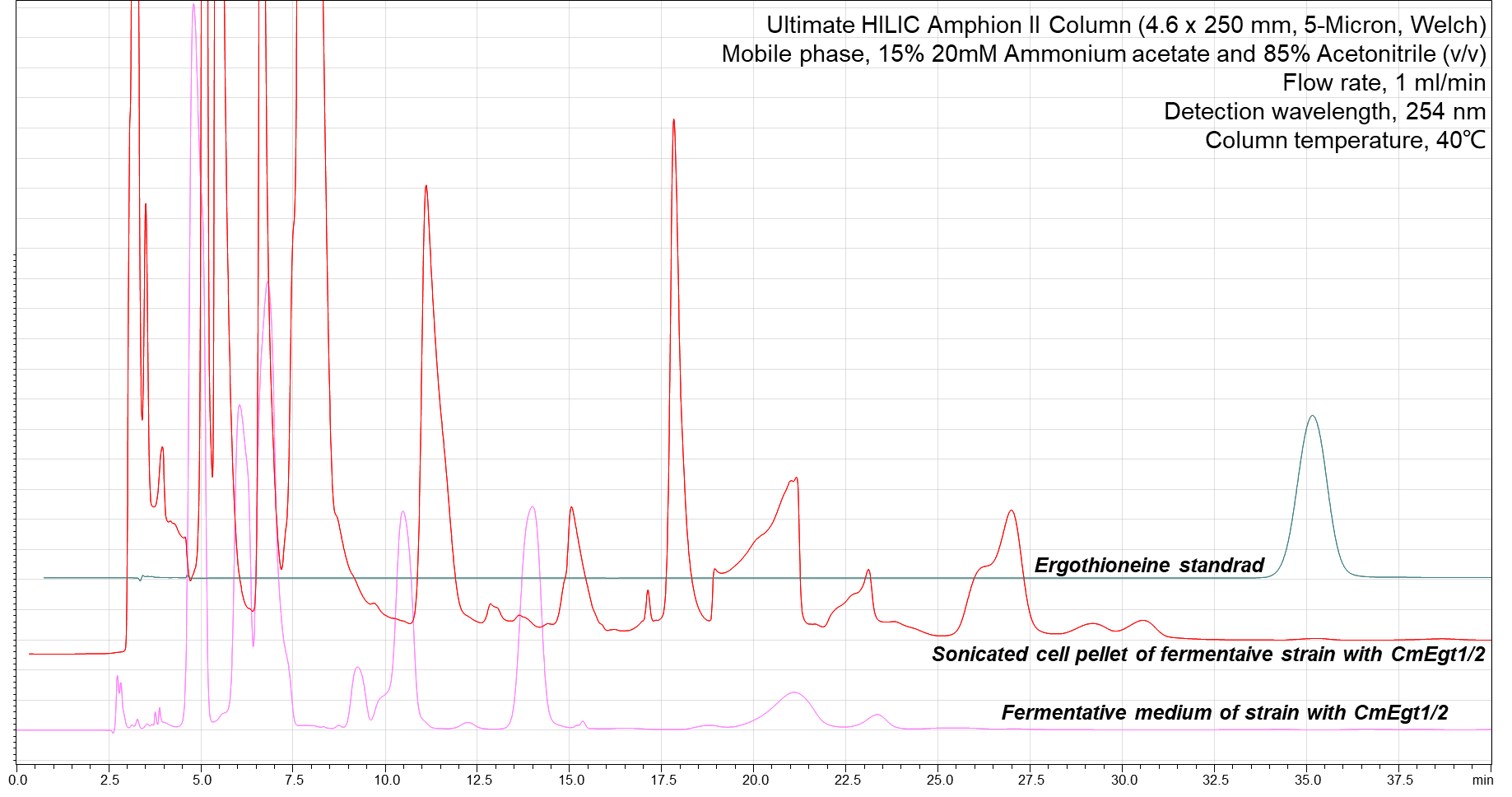


Fig. S1. The HPLC chromatogram of ergothioneine detection of the fermentative medium and cell pellet of BL21-pET-CmEgt1_Egt2.


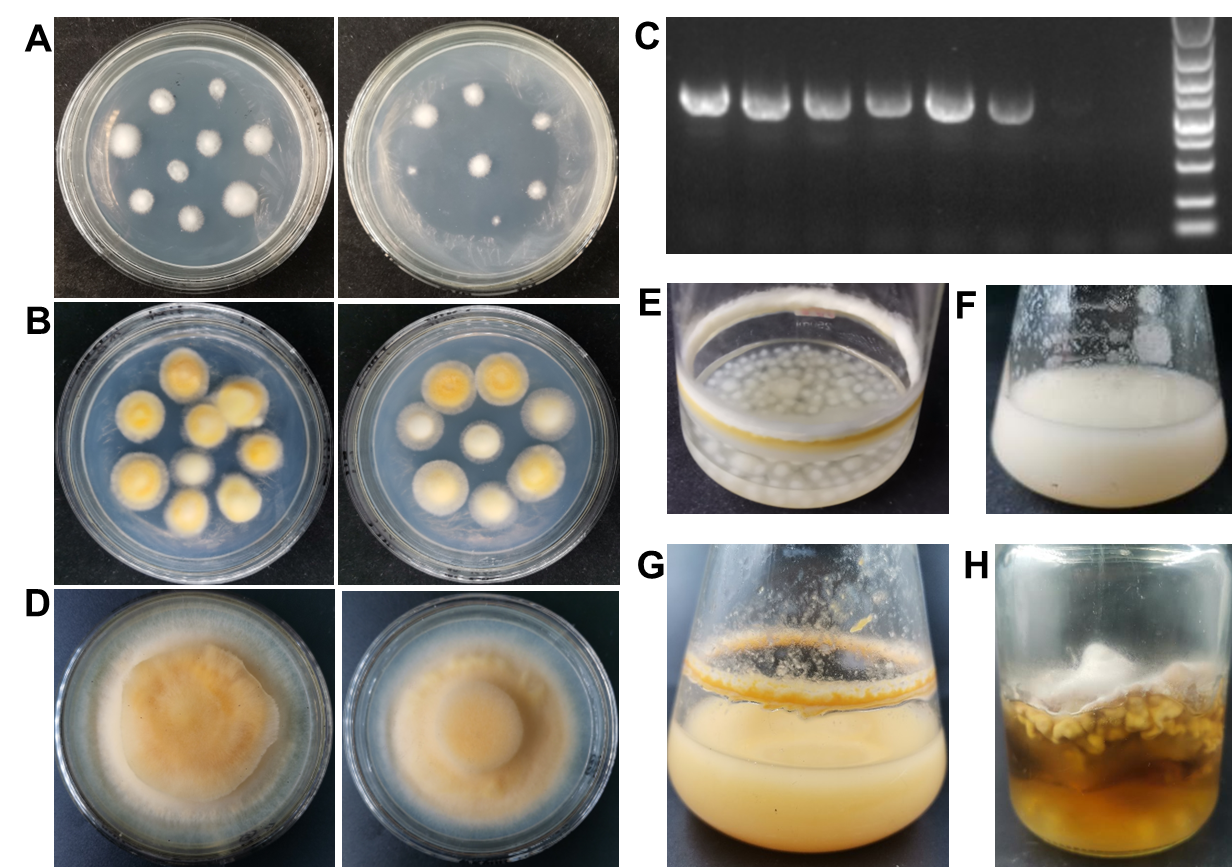


Fig. S2. The verification of qualified recombined C. militaris strains.

(A) Colonies were inculated on resistence screening plates; (B) The remaining colonies were cultivated with alternation of light and dack; (C) Target gene verification of picked strains by PCR; (D) The quilfied recombined strains cultivated on PDA plates were ready to perform fermentation; (E) The healthy status of *C. militairs* cultivated in PDB flask*;* (F) The seed culture of *C. militairs* which were ready to inoculate in fermentative medium; The perfective aspects of *C. militaris* strains which were performing ergothioneine fermentation(G) or cordycepin fermentation(H).
